# Supplementary material for: How insulin-like growth factor I binds to a hybrid insulin receptor type 1 insulin-like growth factor receptor
Source: Structure. 2022 Aug 4;30(8):1098–1108.e6. doi: 10.1016/j.str.2022.05.007 (PMC9364964; doi:10.1016/j.str.2022.05.007)
Supplement: Document S1. Figures S1–S4 and Table S1 [file mmc1.pdf]

Structure, Volume *30*

Supplemental Information

How insulin-like growth factor I binds to a hybrid insulin receptor type 1 insulin-like growth factor receptor

Yibin Xu, Mai B. Margetts, Hari Venugopal, John G. Menting, Nicholas S. Kirk, Tristan I. Croll, Carlie Delaine, Briony E. Forbes, and Michael C. Lawrence

A **Protein Sequence of IR-Bzip**

1	HLYPGEVCPGMDIRNNLTRLHELENCSVIEGHLQILLMFKTRPEDFRDLSFPKLIMITDYLLLFRVYGLESLKDLFPNLT	80
81	VIRGSRLFFNYALVIFEMVHLKELGLYNLMNITRGSVRIEKNNELCYLATIDWSRILDSVEDNYIVLNKDDNEECGDICP	160
161	GTAKGKTNCPATVINGQFVERCWTHSHCQKVCPTICKSHGCTAEGLCCHSECLGNCSQPDDPTKCVACRNFYLDGRCVET	240
241	CPPPYYHFQDWRCVNFSFCQDLHHKCKNSRRQGCHQYVIHNNKCIPECPSGYTMNSSNLLCTPCLGPCPKVCHLLEGEKT	320
321	IDSVTSAQELRGCTVINGSLIINIRGGNNLAAELEANLGLIEEISGYLKIRRSYALVSLSFFRKLRLIRGETLEIGNYSF	400
401	YALDNQNLRQLWDWSKHNLTITQGKLFFHYNPKLCLSEIHKMEEVSGTKGRQERNDIALKTNGDQASCENELLKFSYIRT	480
481	SFDKILLRWEPYWPPDFRDLLGFMLFYKEAPYQNVTEFDGQDACGSNSWTVVDIDPPLRSNDPKSQNHPGWLMRGLKPWT	560
561	QYAIFVKTLVTFSDERRTYGAKSDIIYVQTDATNPSVPLDPISVSNSSSQIILKWKPPSDPNGNITHYLVFWERQAEDSE	640
641	LFELDYCLKGLKLPSRTWSPPFESEDSQKHNQSEYEDSAGECCSCPKTDSQILKELEESSFRKTFEDYLHNVVFVPRKTS	720
721	SGTGAEDPRPSRKRRSLGDVGNVTVAVPTVAAFPNTSSTSVPTSPEEHRPFEKVVNKESLVISGLRHFTGYRIELQACNQ	800
801	DTPEERCSVAAYVSARTMPEAKADDIVGPVTHEIFENNVVHLMWQEPKEPNGLIVLYEVSYRRYGDEELHLCVSRKHFAL	880
881	ERGCRLRGLSPGNYSVRIRATSLAGNGSWTEPTYFYVTDYLDVPSNIARMKQLEDKVEELLSKNYHLENEVARLKKLVGE	960
961	R	

B **Protein Sequence of IGF-1Rzip**

1	EICGPGIDIRNDYQQLKRLENCTVIEGYLHILLISKAEDYRSYRFPKLTVITEYLLLFRVAGLESLGDLFPNLTVIRGWK	80
81	LFYNYALVIFEMTNLKDIGLYNLRNITRGAIRIEKNADLCYLSTVDWSLILDAVSNNYIVGNKPPKECGDLCPGTMEEKP	160
161	MCEKTTINNEYNYRCWTTNRCQKMCPSTCGKRACTENNECCHPECLGSCSAPDNDTACVACRHYYYAGVCVPACPPNTYR	240
241	FEGWRCVDRDFCANILSAESSDSEGFVIHDGECMQECPSGFIRNGSQSMYCIPCEGPCPKVCEEEKKTKTIDSVTSAQML	320
321	QGCTIFKGNLLINIRRGNNIASELENFMGLIEVVTGYVKIRHSHALVSLSFLKNLRLILGEEQLEGNYSFYVLDNQNLQQ	400
401	LWDWDHRNLTIKAGKMYFAFNPKLCVSEIYRMEEVTGTKGRQSKGDINTRNNGERASCESDVLHFTSTTTSKNRIIITWH	480
481	RYRPPDYRDLISFTVYYKEAPFKNVTEYDGQDACGSNSWNMVDVDLPPNKDVEPGILLHGLKPWTQYAVYVKAVTLTMVE	560
561	NDHIRGAKSEILYIRTNASVPSIPLDVLSASNSSSQLIVKWNPPSLPNGNLSYYIVRWQRQPQDGYLYRHNYCSKDKIPI	640
641	RKYADGTIDIEEVTENPKTEVCGGEKGPCCACPKTEAEKQAEKEEAEYRKVFENFLHNSIFVPRPERKRRDVMQVANTTM	720
721	SSRSRNTTAADTYNITDPEELETEYPFFESRVDNKERTVISNLRPFTLYRIDIHSCNHEAEKLGCSASNFVFARTMPAEG	800
801	ADDIPGPVTWEPRPENSIFLKWPEPENPNGLILMYEIKYGSQVEDQRECVSRQEYRKYGGAKLNRLNPGNYTARIQATSL	880
881	SGNGSWTDPVFFYVQAKTGYENFIHRMKQLEDKVEELLSKNYHLENEVARLKKLVGERSSSEQKLISEEDLN	952

Figure S1. Protein sequences of the two constructs use to form HybZip, Related to STAR Methods.

(**A**) IR-Bzip, comprising residues 1-928 of the human IR-B isoform followed by the 33-residue GCN4 zipper sequence (*orange*).

(**B**) IGF-1Rzip, comprising residues 1-905 of human IGF-1R followed by the 33-residue GCN4 zipper sequence (*orange*) followed by a three-serine spacer and the c-myc tag sequence EQKLISEEDLN (*blue*).

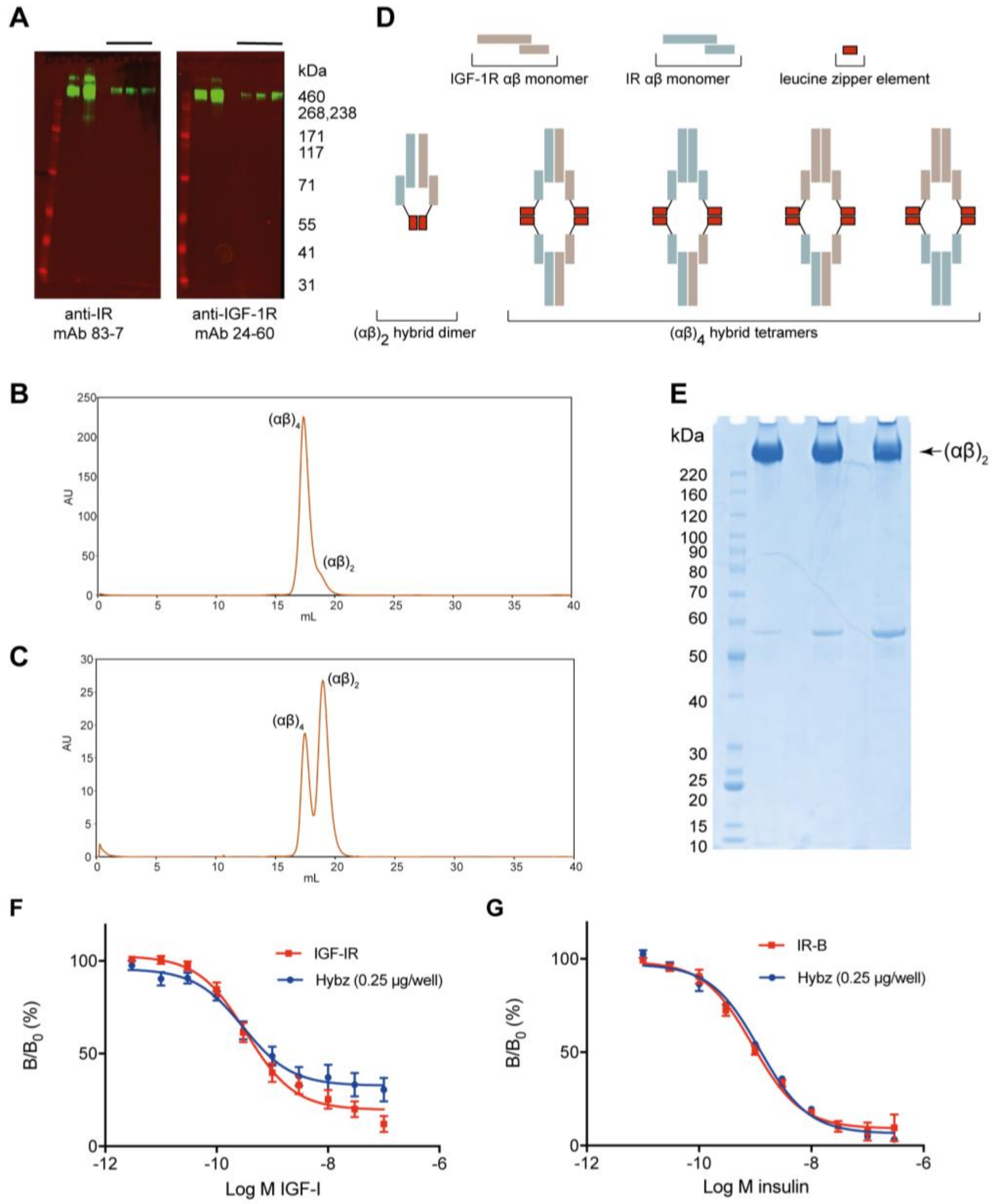

Figure S2. Purification and characterization of HybZip, Related to STAR Methods.

(**A**) Western blots of protein product obtained after serial elution from both 9E10 mAb and 18-44 mAb beads, showing presence of both species in the material (lanes with *bar* on right-hand side of each blot). Left-hand lanes in each blot correspond to pH 3 eluted material obtained from final column wash. Superfluous boundary has been removed from both blot images.

(**B**) Size-exclusion chromatogram obtained from protein product post affinity purification. The dominant peak corresponds to $(\alpha\beta)_4$ species, with a shoulder corresponding to $(\alpha\beta)_2$ species.

(**C**) Enrichment of $(\alpha\beta)_2$ species following re-run of pooled shoulder fractions from (B).

(**D**) Schematic illustrating leucine-zipper cross-linking of receptor ectodomain dimers to form hybrid tetrameric species. All four tetrameric species will be affinity co-purified with the desired hybrid dimer, but can be separated from it by size-exclusion chromatography (see panels B and C).

(**E**) Non-reducing SDS-PAGE gel of pooled $(\alpha\beta)_2$ fractions depicted in (C). Superfluous boundary and blank lanes (to the right) have been removed from the gel image.

(**F**) Competitive displacement assay of labelled IGF-I bound to holo-IGF-1R (n = 9) and to HybZip (n = 12) by unlabelled IGF-I. Error bars reflect standard error of the mean and are omitted when smaller than marker size. B/B_0 = percentage of binding in the absence of competing ligand.

(**G**) Competitive displacement assay of labelled insulin bound to holo-IR-B (n = 9) and to HybZip (n = 9) by unlabelled insulin. Error bars reflect standard error of the mean and are omitted when smaller than marker size. B/B_0 = percentage of binding in the absence of competing ligand.

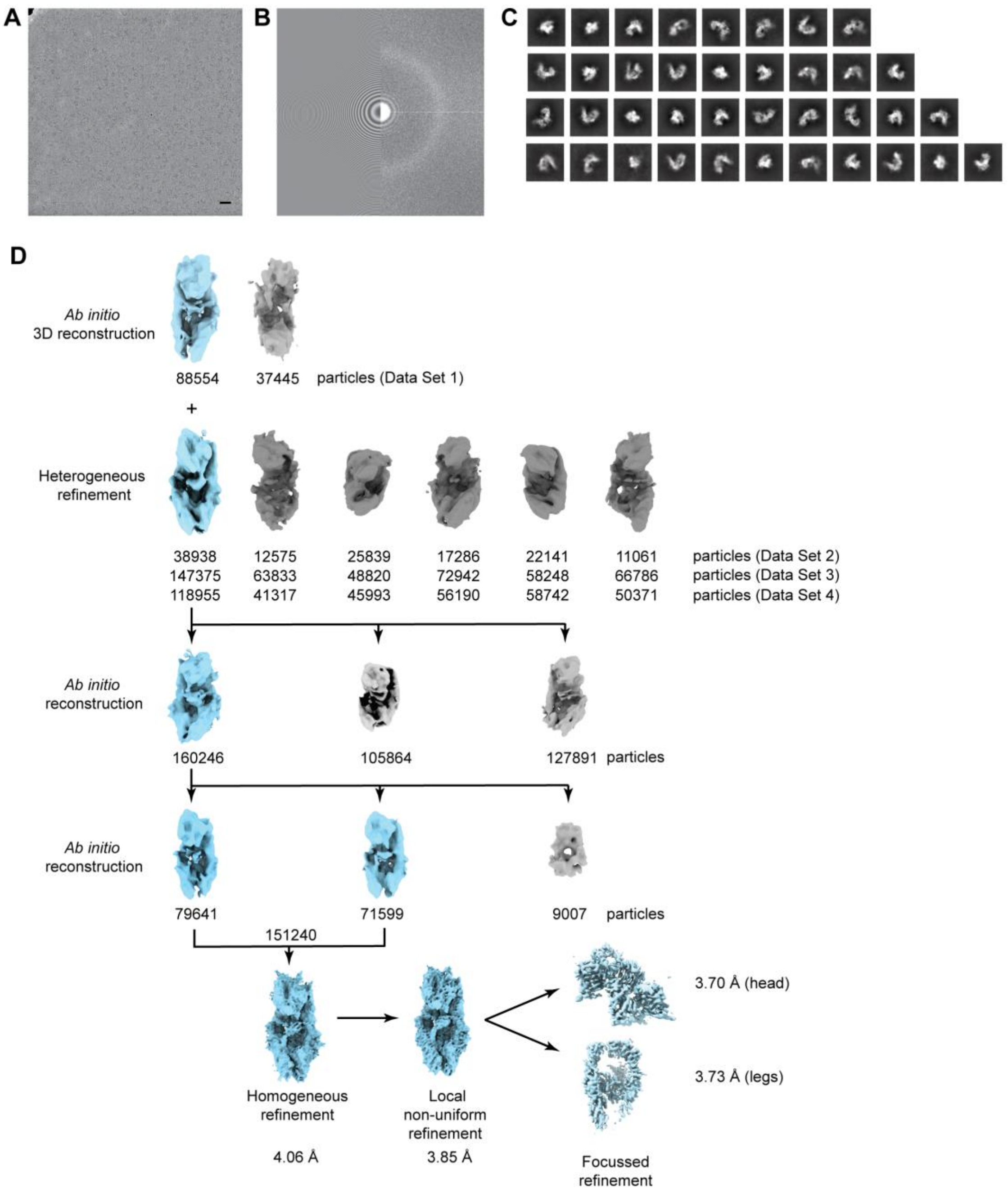

Figure S3. CryoEM reconstruction of IGF-I-complexed HybZip, Related to STAR Methods.

(**A**) Representative image obtained after motion correction (scale bar = 200 Å)

(**B**) Contrast transfer function associated with (A).

(**C**) 2D classes of particles selected from the four data sets (one data set per row; individual class images have been cropped to facilitate enlarged display).

(**D**) Flowchart showing the process of 3D reconstruction of IGF-I-complexed HybZip.

Full details are provided in the **STAR Methods Detail** section.

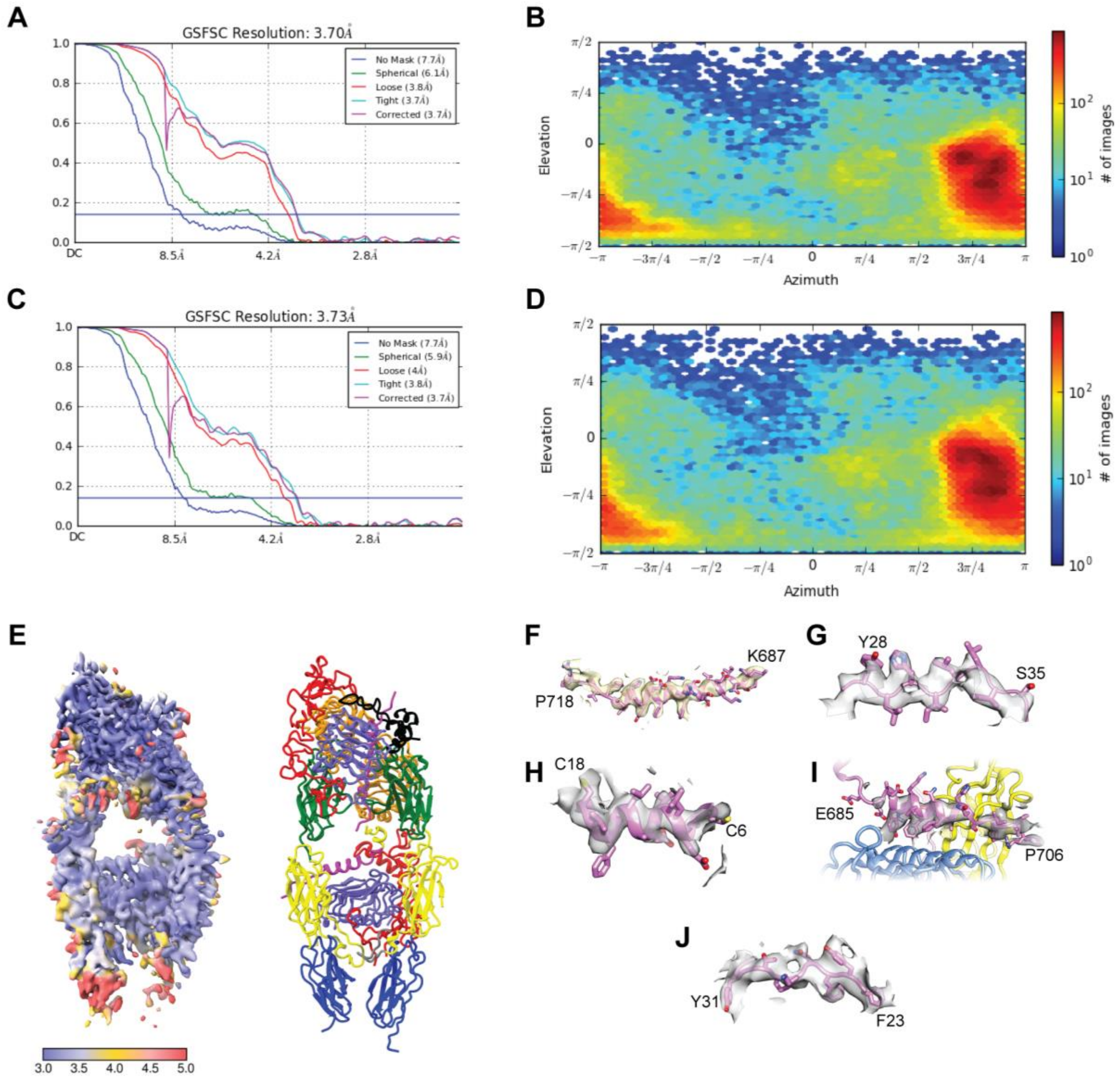

Figure S4. Quality of the 3D reconstructions of the two receptor volumes, Related to STAR Methods.

(**A**),(**C**) Gold-standard Fourier shell correlation (GSFSC) plots for the half-maps associated with the reconstruction of the "head" and "leg" region, respectively, of the IGF-I-complexed HybZip.

(**B**), (**D**) Angular distribution of particles contributing to the reconstruction of the "head" and "leg" region, respectively, of the IGF-I-complexed HybZip.

(**E**) Left panel: overlay of the "head" and "leg" regions maps, colored according to the local resolution. Right panel: ribbon diagram of IGF-I-complexed HybZip, oriented and scaled to match the local-resolution map in the left panel.

(**F**) CryoEM potential density for IR αCT segment, residues Lys687-Pro718. Contour level 0.201, display restricted to within 3.0 Å of depicted residues.

(**G**) CryoEM potential density for IGF-1R domain L1, residues Tyr28-Ser35. Contour level 0.230, display restricted to within 3.0 Å of depicted residues.

(**H**) CryoEM potential density for B domain helix of IGF-I, residues Cys6-Cys18. Contour level 0.242, display restricted to within 3.0 Å of depicted residues.

(**I**) CryoEM potential density for IGF-1R αCT segment, residues Glu685-Pro706. Contour level 0.201, display restricted to within 3.4 Å of depicted residues.

(**J**) CryoEM potential density for C domain of IGF-I, residues Phe23-Tyr31. Contour level 0.160, display restricted to within 3.0 Å of depicted residues.

Table S1. CryoEM data collection parameters, related to STAR METHODS

Data Set:	1	2	3	4
Pixel size (Å)	1.06	1.06	1.06	1.06
Dose rate ($e^{-}.pix^{-1}.sec^{-1}$)	8	6	6	6
Total dose ($e^{-}.Å^{-2}$)	50	52	52	52
No. frames/movie	36	50	50	50
C_s	2.7	2.7	2.7	2.7
kV	300	300	300	300
