# Supplementary material for: How insulin-like growth factor I binds to a hybrid insulin receptor type 1 insulin-like growth factor receptor
Source: Structure. 2022 Aug 4;30(8):1098–1108.e6. doi: 10.1016/j.str.2022.05.007 (PMC9364964; doi:10.1016/j.str.2022.05.007)
Supplement: Document S2. Article plus supplemental information [file mmc2.pdf]

Structure

Article

How insulin-like growth factor I binds to a hybrid insulin receptor type 1 insulin-like growth factor receptor

Graphical abstract

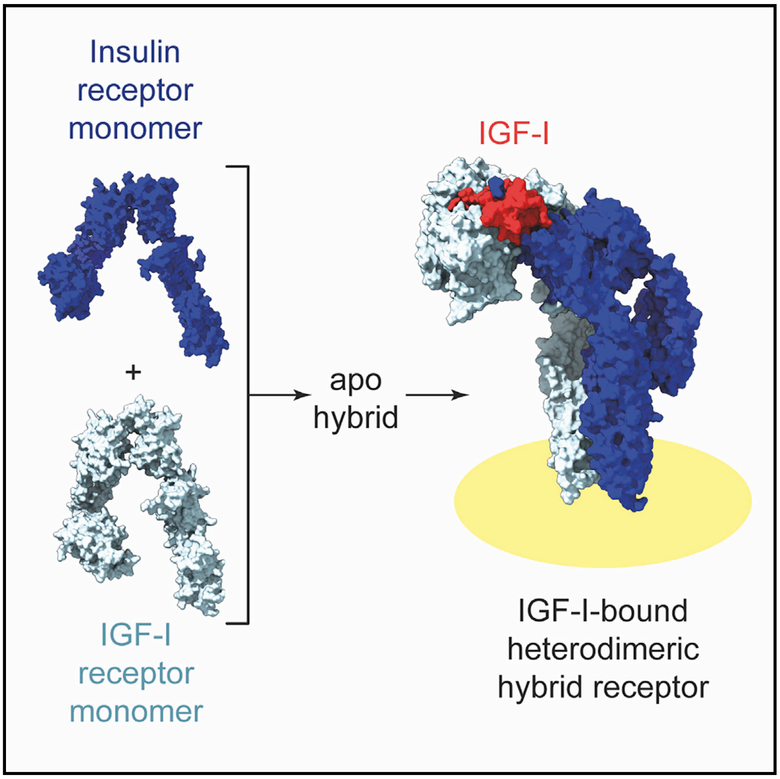

Highlights

- A cryo-EM structure of IGF-I bound to a hybrid IR/IGF-1R ectodomain is presented
- The structure is congruent to those of the single-liganded homodimeric receptors

Authors

Yibin Xu, Mai B. Margetts, Hari Venugopal, ..., Carlie Delaine, Briony E. Forbes, Michael C. Lawrence

Correspondence

lawrence@wehi.edu.au

In brief

Monomers of the insulin- and type 1 insulin-like growth factor receptors are capable of forming dimeric, disulphide-linked hybrid receptors. Xu et al. show that such hybrid receptors bind IGF-I in a fashion similar to that of the parent IGF-I receptor and to that of the single-liganded insulin receptor.

Xu et al., 2022, Structure *30*, 1098–1108
August 4, 2022 © 2022 The Author(s). Published by Elsevier Ltd.
https://doi.org/10.1016/j.str.2022.05.007

CellPress
OPEN ACCESS

Structure

Article

How insulin-like growth factor I binds to a hybrid insulin receptor type 1 insulin-like growth factor receptor

Yibin Xu,[1,2] Mai B. Margetts,[1] Hari Venugopal,[3] John G. Menting,[1,2] Nicholas S. Kirk,[1,2] Tristan I. Croll,[4] Carlie Delaine,[5] Briony E. Forbes,[5] and Michael C. Lawrence[1,2,6,*]

[1]WEHI, 1G Royal Parade, Parkville, VIC 3052, Australia
[2]Department of Medical Biology, Faculty of Medicine, Dentistry and Health Sciences, University of Melbourne, Parkville, VIC 3050, Australia
[3]Ramaciotti Centre for Cryo-Electron Microscopy, Monash University, Clayton, VIC 3800, Australia
[4]Cambridge Institute for Medical Research, University of Cambridge, Keith Peters Building, Cambridge CB2 0XY, UK
[5]Discipline of Medical Biochemistry, College of Medicine and Public Health, Flinders University of South Australia, Bedford Park, SA 5042, Australia
[6]Lead contact
*Correspondence: lawrence@wehi.edu.au
https://doi.org/10.1016/j.str.2022.05.007

SUMMARY

Monomers of the insulin receptor and type 1 insulin-like growth factor receptor (IGF-1R) can combine stochastically to form heterodimeric hybrid receptors. These hybrid receptors display ligand binding and signaling properties that differ from those of the homodimeric receptors. Here, we describe the cryoelectron microscopy structure of such a hybrid receptor in complex with insulin-like growth factor I (IGF-I). The structure (ca. 3.7 Å resolution) displays a single IGF-I ligand, bound in a similar fashion to that seen for IGFs in complex with IGF-1R. The IGF-I ligand engages the first leucine-rich-repeat domain and cysteine-rich region of the IGF-1R monomer (rather than those of the insulin receptor monomer), consistent with the determinants for IGF binding residing in the IGF-1R cysteine-rich region. The structure broadens our understanding of this receptor family and assists in delineating the key structural motifs involved in binding their respective ligands.

INTRODUCTION

The insulin receptor (IR) and the type 1 insulin-like growth factor receptor (IGF-1R) are two closely related, homodimeric $(\alpha\beta)_2$ receptor tyrosine kinases. Insulin signaling via IR effects glucose homeostasis as well as being implicated in lipogenesis, protein synthesis, cell growth, cell differentiation, and neuroplasticity (Saltiel and Kahn, 2001; Taniguchi et al., 2006; Arnold et al., 2018). IGF-I and IGF-II signal via IGF-1R, playing roles in normal human growth (Chitnis et al., 2008). Aberrant signaling by these receptors is implicated in the disease states of diabetes, cancer, and Alzheimer's disease. Intriguingly, IR and IGF-1R αβ monomers are capable of forming signaling-competent hybrid IR/IGF-1R heterodimers in tissues that express both receptors. Within these hybrids, one αβ monomer derives from IR and the other from IGF-1R (Soos and Siddle, 1989; Moxham et al., 1989; Soos et al., 1990; Bailyes et al., 1997). Evidence exists that the ratio of homodimeric to heterodimeric receptors in certain tissues is determined stochastically as a function of the level of expression of the individual receptors within a particular cell, with the efficiency of αβ heterodimerization being equivalent to that of homodimerization (Bailyes et al., 1997). The degree of hybrid receptor formation may also be modulated in other ways in specific tissues (Bailyes et al., 1997; Mughal et al., 2018). A distinct physiological role for the hybrid receptors is unclear, as ligand binding to the hybrid receptors induces phosphorylation of both receptor halves (Belfiore et al., 2017). Hybrid receptors, like their homodimeric counterparts, have been shown to translocate to the nucleus (Titone et al., 2018).

IR itself has two splice-variants—termed IR-A and IR-B, with IR-A lacking and IR-B retaining the 12-residue polypeptide product of exon 11 (Seino and Bell, 1989). Both the IR-A αβ and IR-B αβ monomers are capable of heterodimerizing with IGF-1R, forming, respectively, IR-A/IGF-1R and IR-B/IGF-1R hybrid receptors (Pandini et al., 2002). The two IR isoforms hybridize with IGF-1R with similar efficiency (Belfiore, 2007).

Of the three ligands (insulin, IGF-I, and IGF-II) in the interconnected homodimeric heterodimeric receptor signaling systems, all are capable of binding to all forms of the receptors, though each displays highest activity with respect to their cognate homodimeric receptor (Belfiore et al., 2017). IGF-I's activity with respect to hybrid receptors is similar to (or somewhat stronger than) that of IGF-II, and its activity with respect to the IR-A/IGF-1R hybrid receptor is similar to (or somewhat stronger than) that with respect to IR-B/IGF-1R (Belfiore et al., 2017). Insulin's activity with respect to hybrid receptors is at least one to two orders of magnitude weaker than that of the IGFs (Belfiore et al., 2017) and, as such, is probably physiologically irrelevant.

 © 2022 The Author(s). Published by Elsevier Ltd.
This is an open access article under the CC BY license (http://creativecommons.org/licenses/by/4.0/).

CellPress
OPEN ACCESS

No structural data exist for the complete ectodomain of the hybrid receptor in either apo or ligand-bound form. Three-dimensional structures—derived from both X-ray crystallography and single-particle cryoelectron microscopy (cryo-EM) studies—have nevertheless revealed the manner in which insulin and the IGFs bind to the homodimeric receptor ectodomains and effect the conformational changes that result in signal transduction and transactivation of the receptor (McKern et al., 2006; Smith et al., 2010; Menting et al., 2013, 2014; Croll et al., 2016; Scapin et al., 2018; Gutmann et al., 2018, 2020; Weis et al., 2018; Xu et al., 2018, 2020; Uchikawa et al., 2019; Li et al., 2019; Zhang et al., 2020). These structural data can be summarized briefly as follows: in the apo form, the receptor ectodomains have a 2-fold-symmetric, Λ-shaped conformation, brought about by juxtaposition of the L1-CR-L2 module of each receptor monomer with the [FnIII-1]-[FnIII-2]-[FnIII-3] module of the opposing monomer (McKern et al., 2006; Smith et al., 2010; Croll et al., 2016; Gutmann et al., 2018; Xu et al., 2018), using the receptor domain nomenclature defined in McKern et al. (2006) (see caption to Figure 1A). Within this assembly, the C-terminal segment (αCT) of each receptor α chain associates with the L1 domain of the opposite monomer, with this tandem element forming the "primary" ligand-binding site (Smith et al., 2010; Menting et al., 2013, 2014, 2015; Xu et al., 2018; Xiong et al., 2020). Ligand engagement with this primary site results in a large conformational change within the L1-CR-L2 module that transports the ligand-bound L1+αCT' tandem element to the apex of the receptor and brings the ligand into contact with the membrane-distal loops of domain FnIII-1' (with the prime symbols denoting structural elements contributed by the opposing $\alpha\beta$ monomer to that which contributes domain L1) (Scapin et al., 2018; Weis et al., 2018; Uchikawa et al., 2019; Li et al., 2019; Xu et al., 2020; Gutmann et al., 2020; Zhang et al., 2020). Concomitant change also occurs in the αCT' element (namely, its rearrangement on the L1 domain surface and its N- and C-terminal helical extension) as well as in the FnIII domain modules, with the latter being brought into juxtaposition at the membrane-proximal region of the ectodomain. In the cryo-EM structures of insulin-liganded IR, insulin-to-receptor stoichiometry is seen as either 1:1, 2:1, or 4:1, likely arising from variations in the stoichiometric mixes used for the cryo-EM grid preparations by different research teams. The [two-insulin]-bound structure has the pair of insulin ligands bound symmetrically (or pseudo-symmetrically) to the respective primary sites, with each insulin interacting further (albeit sparsely) with the membrane-distal loops of the opposing domains FnIII-1 (Scapin et al., 2018). The [four-insulin]-bound IR structures have two additional insulins each bound to a "secondary" site on the respective outward-facing surfaces of domains FnIII-1 and FnIII-1' (Uchikawa et al., 2019; Gutmann et al., 2020). The role of these latter sites is uncertain, but they may function as transient insulin-binding sites that enable exposure of the otherwise partly occluded primary binding sites (Uchikawa et al., 2019; Lawrence, 2021). In contrast, cryo-EM structures of IGF-I- and IGF-II-complexed IGF-1R have displayed only a single bound ligand (Li et al., 2019; Xu et al., 2020; Zhang et al., 2020) and are overall conformationally similar to the [single-insulin]-bound structure of IR (Weis et al., 2018).

Study of chimeric receptors has shown that IGF-1R specificity for IGFs resides in the receptor's cysteine-rich region (Schumacher et al., 1991). IGF-I displays an order-of-magnitude higher affinity for a hybrid "mini-receptor" comprising the L1-CR-L2 module of IGF-1R and the αCT segment of IR than for a hybrid "mini-receptor" comprising the L1-CR-L2 module of IR and the αCT segment of IGF-1R (Kristensen et al., 1999). Together, these data suggest that in the intact hybrid receptor context, IGFs will bind preferentially to the tandem element assembled from the IGF-1R L1-CR module and the IR αCT segment rather than to the tandem element assembled from the IR L1-CR module and the IGF-1R αCT segment. Here, we have implicitly assumed that the association of the αCT elements and the L1-CR modules will be in *trans* (Smith et al., 2010), i.e., that, within the mature hybrid receptor, the αCT element of one receptor monomer is located on the surface of domain L1 of the other receptor monomer. However, no structural data are available to confirm this within the hybrid receptor. The only structural information relevant to hybrid receptors is the structure of IGF-I in co-complex with the isolated L1-CR module of IR and the αCT peptide of IGF-1R (Menting et al., 2015), which may not thus reflect the physiological engagement of IGF-I with intact hybrid receptor given that it is the alternate pair of receptor elements.

To endeavor to resolve these issues and to understand the mode of assembly of the hybrid receptor and its mode of ligand engagement, we present here a single-particle cryo-EM structure of IGF-I in complex with an IR-B/IGF-1R hybrid receptor. We show that the overall conformation of the membrane-distal region of the IGF-I-bound region of the hybrid receptor is closely similar to that seen for IGF-I and IGF-II complexes of the IGF-1R homodimeric receptor (Li et al., 2019; Xu et al., 2020). Notably, only a single ligand is observed bound to the hybrid receptor despite the complex being prepared in a 4-fold molar excess of IGF-I. The structure is thus distinct from those of insulin in complex with the IR, wherein varying ligand-to-receptor stoichiometry is observed (Gutmann et al., 2020; Li et al., 2019; Nielsen et al., 2022).

RESULTS

Production and purification of the HybZip

The receptor constructs employed here to generate the IR-B/IGF-1R hybrid receptor comprise the respective receptor ectodomain monomers, each C terminally extended by a leucine-zipper element (O'Shea et al., 1991) (Figure S1). Leucine-zipper attachment has been shown to restore holo-receptor-like properties to the isolated IR ectodomain (Hoyne et al., 2000), and its value in cryo-EM has been demonstrated in both the generation of a cryo-EM structure of the IR-A ectodomain in complex with insulin (Weis et al., 2018) and of the IGF-1R ectodomain in complex with IGF-II (Xu et al., 2020). The zipper arguably acts as a mimic of membrane anchoring and likely lends structural stability to the isolated ectodomain. Here, the zippered ectodomain of the hybrid receptor (termed HybZip) was produced via stable co-expression of both constructs in zippered form (IR-Bzip and IGF-1Rzip, respectively), with cell-line selection being guided by the requirement of qualitatively similar levels of individual receptor expression. Purification of HybZip from conditioned media was achieved by sequential

antibody affinity chromatography using monoclonal antibody (mAb) 9E10 resin directed against a c-myc tag attached to IGF-1Rzip (Hilpert et al., 2001; Xu et al., 2020) followed by mAb 18-44 resin directed against a linear epitope within the N-terminal region of the IR-B β chain (Soos et al., 1986; Prigent et al., 1990). mAb 18-44 does not cross-react with IGF-1R (Soos et al., 1990; Zhang and Roth, 1991). Western-blot analysis using mAb 83-7 (specific for IR; Soos et al., 1986) and mAb 24-60 (specific for IGF-1R; Soos et al., 1992) confirmed the presence of both receptor species in the affinity-purified protein product (Figure S2A). Size-exclusion chromatography was then used to remove aberrant $(\alpha\beta)_4$ fractions from the desired heterodimeric $(\alpha\beta)_2$ fraction (Weis et al., 2018; Xu et al., 2020) (Figures S2B and S2C). Similar mixtures of dimeric and tetrameric receptor species have been seen in related studies that employ zipper-stabilized ectodomains (Hoyne et al., 2000; Weis et al., 2018; Xu et al., 2020), with the tetrameric species being solely an artifact of zipper attachment (Figure S2D). The final protein product HybZip was visualized as a single band of the anticipated molecular weight on a non-reducing SDS gel (Figure S2E). Full details of production and purification of HybZip are presented in the STAR methods.

Characterization of HybZip

IGF-I bound to the purified HybZip with a half-maximal inhibitory concentration (IC_{50}) of 0.30 nM (95% confidence interval: 0.17–0.54 nM) in a competition assay displacing europium-labeled IGF-I, compared with an IC_{50} of 0.34 nM (95% confidence interval: 0.23–0.49 nM) in an equivalent assay directed toward holo-IGF-1R (one-site fit; Figure S2F). This affinity of the hybrid receptor for IGF-I is similar to that determined previously using I^{125}-labeled IGF-I (Pandini et al., 2002; Benyoucef et al., 2007). Insulin bound the purified HybZip with an IC_{50} of 1.2 nM (95% confidence interval: 1.0–1.4 nM) in a competition assay displacing europium-labeled insulin, compared with an IC_{50} of 0.90 nM (95% confidence interval: 0.67–1.20 nM) in an equivalent assay directed toward holo-IR-B (one-site fit; Figure S2G). Whereas there are no previous reports of binding studies using labeled insulin, this affinity for the hybrid receptor is similar to those determined previously using I^{125}-labeled IGF-I (Slaaby et al., 2006). We accept that the IC_{50} values cited above may be affected by residual tetramer components within the purified product.

Single-particle cryo-EM

Purified HybZip was combined in a 1:4 mole ratio with human IGF-I and then subjected to cryo-EM imaging at 300 kV, with movies being collected in four separate data sets. Particles were selected independently from the four data sets based on the identification of well-defined 2D classes. 3D reconstruction began with a low-resolution *ab initio* 3D reconstruction from the first data set, which was then used to inform 3D classification of the combined particles of the remaining data sets. The combined 393,822 particles from all four data sets were further cleaned up by two rounds of *ab initio* reconstruction—each into three classes; the process led to the final 151,240 particles (Figure S3), permitting reconstruction to an average resolution of ca. 3.85 Å. The retained 3D class was then further split into “head” and “leg” regions to allow focused refinement of each map volume, achieving an average respective resolution of 3.70 and 3.73 Å for the two regions. Resolution estimates were based on the 0.143 cut-off criterion in the gold-standard Fourier-shell correlation (GS-FSC) coefficient of the two independent half maps (Figures S4A and S4C). Whereas there was some evidence of anisotropy in the data sets (Figures S4B and S4D), this was not judged as severe. Local resolution estimates (Figure S4E) were commensurate with that based on the GS-FSC assessment. Sample potential density for regions of interest of the maps are given in Figures S4F–S4J. The strategy of separately focus-refining the head- and leg regions of the receptor mimics that employed for the insulin-bound IRΔβ-zip structure (Weis et al., 2018) and the [IGF-II]-bound IGF-1Rzip structure (Xu et al., 2020). Full details of the cryo-EM image processing and reconstruction pipeline are presented in the STAR methods.

Overview of the structure

The [IGF-I]-bound HybZip (Figure 1A) was readily discerned to have similar overall domain organization to that visualized for the [IGF-I]-bound holo-IGF-1R (Li et al., 2019), insulin-bound IRΔβzip (Weis et al., 2018), [IGF-II]-bound IGF-1R.zip (Xu et al., 2018), and insulin-bound IGF-1R (Zhang et al., 2020). Only a single IGF-I moiety was detected within the structure, despite their 4:1 mole ratio in the cryo-EM sample. However, the limited resolution of the cryo-EM maps (Figures 1B and 1C)—combined with the high level of sequence similarity between IGF-1R and IR (McKern et al., 2006)—precluded discrimination of the respective chains based on residue side-chain density alone. Nevertheless, a clear discriminator occurred within the sixth structural module of the respective CR domains—these modules span IR residues 255–286 and IGF-1R residues 248–275, respectively (Lou et al., 2006). In IR, the module contains a single α helix spanning ca. 15 residues, whereas in IGF-1R, the equivalent helix spans only ca. 6 residues (Lou et al., 2006). The respective lengths of these helices are maintained in all extant structures of the two receptors, regardless of their apo or liganded state (Figure 1D). In our map, we observed the longer helix within the [IGF-I]-free L1-CR module and the shorter helix within the [IGF-I]-bound L1-CR module, allowing assignment of the former as arising from IR and the latter from IGF-1R (Figure 1D).

As mentioned above, the structure described here is similar to those of the single-liganded forms of the corresponding homodimeric receptors. Overlay of the respective receptor head regions reveals only limited relative domain displacement and rotation (Figures 2A–2D). However, more substantial variation is seen across the respective receptor leg regions (Figures 2E–2H), where varying rotation and displacement of pairs of FnIII domains leads in turn to varying distances between the points of membrane insertion. Also apparent across the suite of structures is the varying way in which the αCT segment engages the ligand-free domain L1. Within the current structure, inter-monomer contacts form between the respective α-chain insert domain components of the receptor monomers, and IR domain CR’ is in contact with the adjacent IR domain FnIII-2’, appearing to preclude contact between the membrane-proximal FnIII-3 domains (see, e.g., in Figure 2E). However, the points of membrane entry of the FnIII-3 domains are closer together here (ca. 27 Å) than in [IGF-I]-bound holo-IGF-1R (ca. 40 Å; Figure 2E) but further apart than in insulin-bound IRΔβ.zip (ca. 15 Å; Figure 2G). The

CellPress
OPEN ACCESS

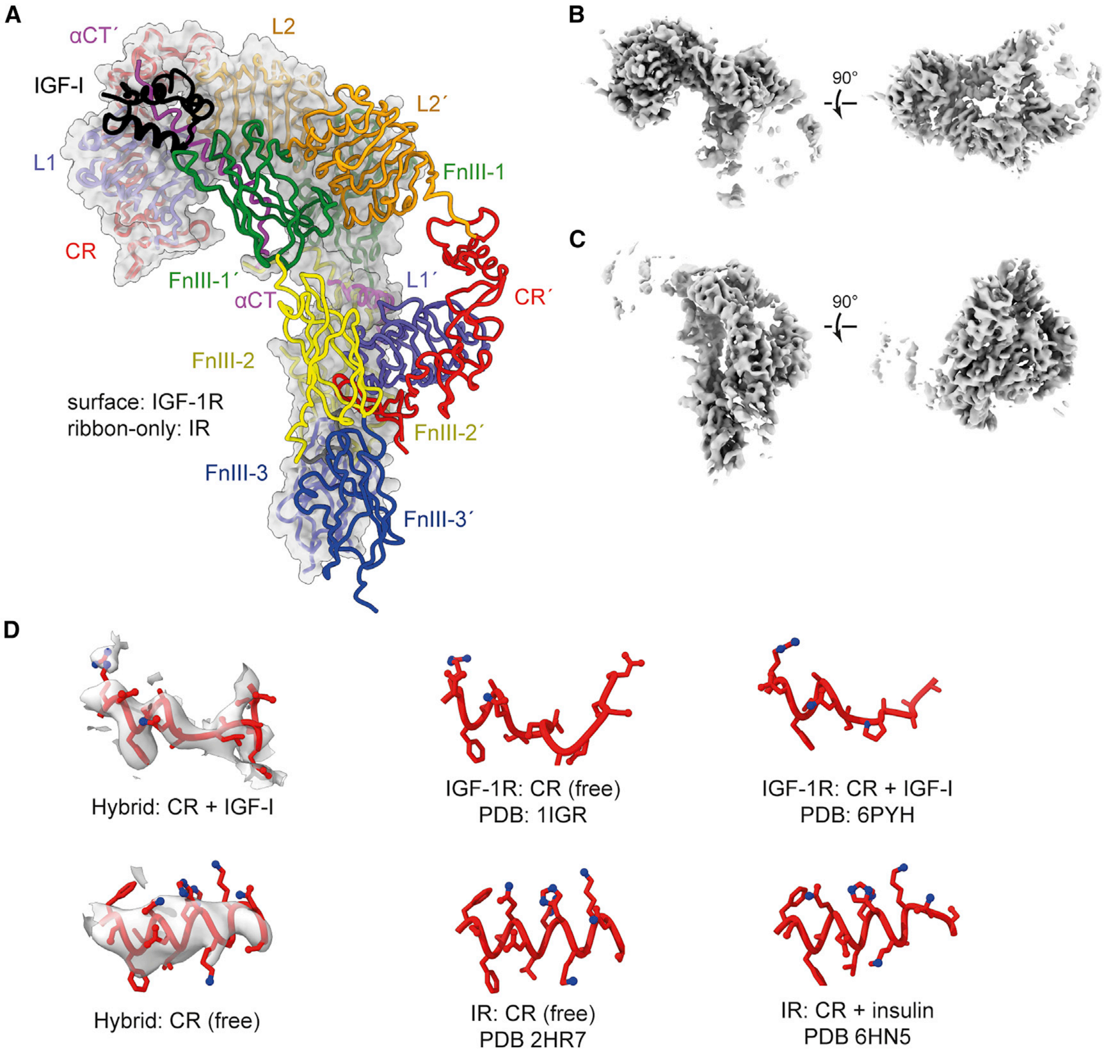

Figure 1. Overview of the structure of [IGF-I]-complexed HybZip

(A) Overall conformation of [IGF-I]-complexed HybZip. Domain nomenclature: L1, first leucine-rich repeat domain; CR, cysteine-rich region; L2, second leucine-rich repeat domain; FnIII-1,-2,-3, first, second, and third fibronectin type III domain; αCT, C-terminal segment of the receptor α chain (lying within the insert domain ID). IGF-1R monomer domains are in transparent molecular surface plus ribbon representation, IR monomer domains are in ribbon-only representation denoted with a prime (') symbol. IGF-I is in black ribbon.

(B) Coulombic potential associated with the head region of the structure.

(C) Coulombic potential associated with the leg region of the structure.

(D) Identification of monomers within [IGF-I]-complexed HybZip on the basis of the N-terminal α helix lying within the sixth module of their respective CR domains compared with the structures of this helical element within selected extant structures of the receptors.

significance of these latter differences is unclear, as they may arise from (1) genuine structural differences across the receptor family, (2) the varied nature of the constructs employed, (3) variation in the cryo-EM sample preparation, and/or (4) inadvertent selection of one of multiple co-existing conformations in the sample. In particular, we note that variation in the relative displacement between the points of membrane entry is observed within the extant suite of insulin-complexed IR structures (Lawrence, 2021), suggesting that the source is sample dependent. Ultimately, however, the varying displacements may indirectly reflect subtleties in signaling.

Given the lack of structural resolution, it is not clear whether the two αCT segments associate in *trans* or in *cis* with the respective HybZip L1 domains, as the sequence similarity of the αCT segment across the receptors is very high, and it is not possible to discern in the cryoEM map the residues corresponding to the exon 11 component of IR-B. Nevertheless, photo-cross-linking of insulin to holo-IR has shown the association to be *trans* (Smith et al., 2010), and we have hence chosen to assume below that the αCT segments are here too in *trans* association with the respective domains L1.

The mode of IGF-I binding

IGF-I interacts with IGF-1R domains L1 and CR and with IR domain FnIII-1 and the IR αCT segment (Figure 3A). The IR αCT segment, corresponding to that of IR-B, "threads" through the C domain of

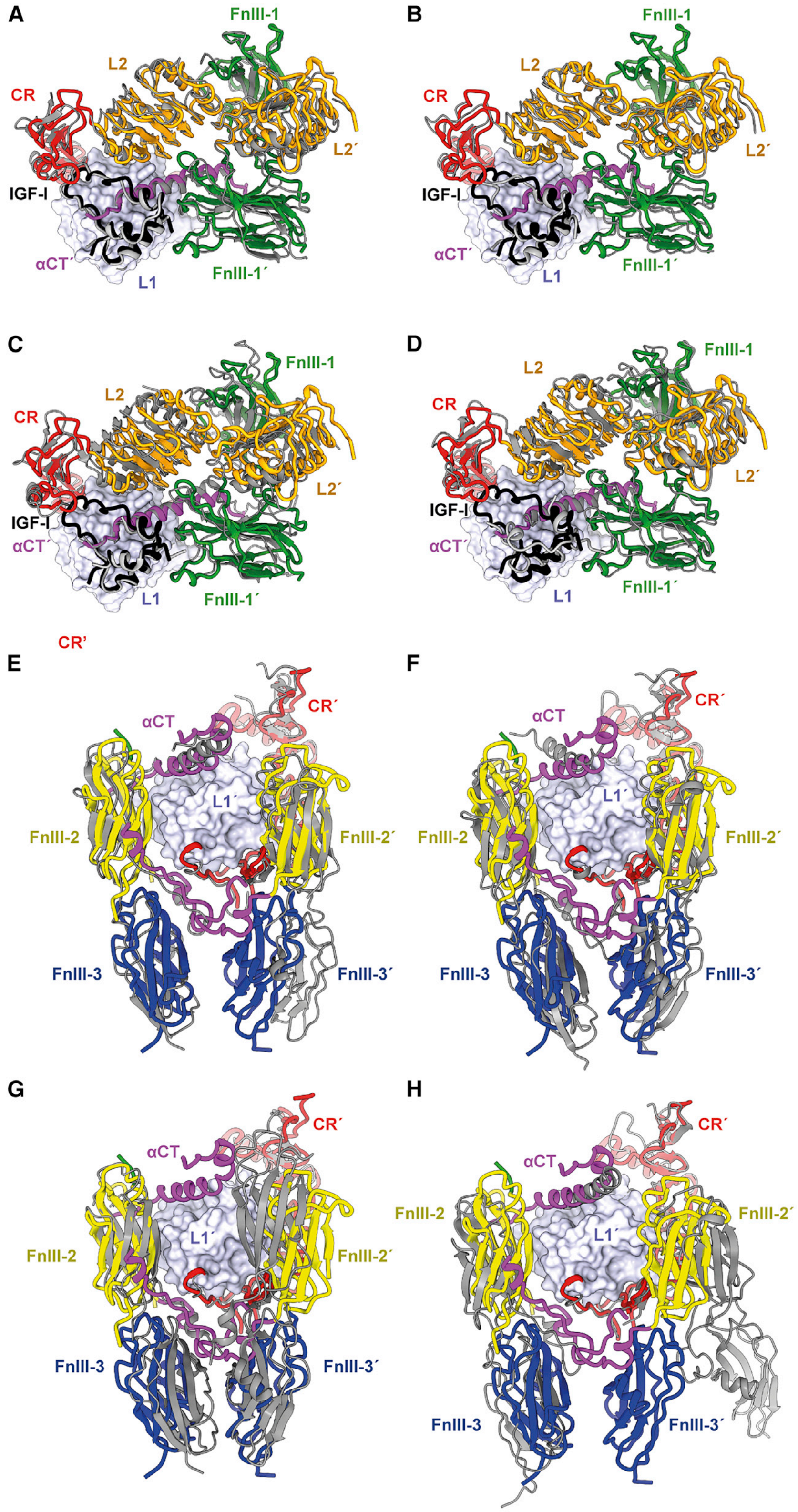

Figure 2. Overlay of the [IGF-I]-bound HybZip structure onto the single-liganded structures of the corresponding homodimer receptors

(A and E) Overlay of [IGF-I]-bound HybZip onto [IGF-I]-bound IGF-1R (PDB: 6PYH).

(B and F) Overlay of [IGF-I]-bound HybZip onto [IGF-II]-bound IGF-1R (closed-leg form; PDB: 6VWI).

(C and G) Overlay of [IGF-I]-bound HybZip onto [single-insulin]-bound IR (PDB: 6HN5).

(D and H) Overlay of [IGF-I]-bound HybZip onto insulin-bound IGF-1R (PDB: 6JK8).

In all panels, [IGF-I]-bound HybZip is displayed as colored ribbon, and the liganded homodimeric receptor is displayed as gray ribbon. Overlays are based on the common domain L1 (light blue surface).

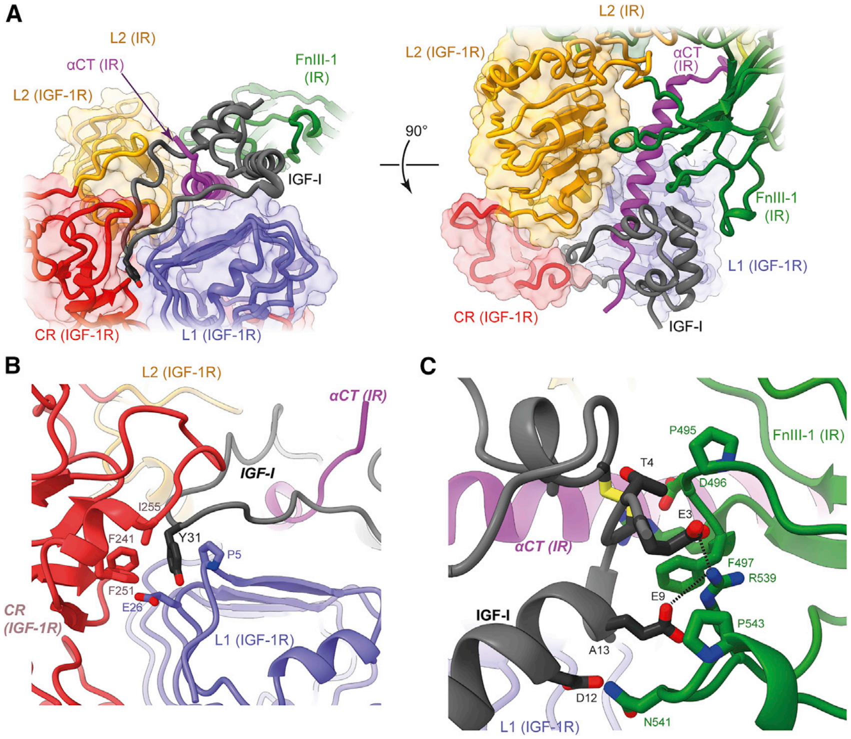

Figure 3. Detail of the structure of [IGF-I]-complexed HybZip

(A) Orthogonal views of IGF-I engagement with surrounding receptor domains.

(B) Engagement of IGF-I residue Tyr31 with a pocket at the junction of domains L1 and CR of the IGF-1R monomer.

(C) Engagement of IGF-I with domain FnIII-1 of the IR monomer.

IGF-I. Such threading of an IGF ligand has not been shown previously for a "long" αCT segment, as all prior [IGF-I]- and [IGF-II]-complexed receptor structures involve IGF-1R (Li et al., 2019; Xu et al., 2020), which has a 12-residue-shorter αCT segment than that of IR-B (Ullrich et al., 1986; Seino and Bell, 1989).

Within this structure, the IGF-I C domain appears anchored to the receptor via IGF-I residue Tyr31, the side chain of which engages a pocket formed by the side chains of IGF-1R residues Pro5, Glu26, Phe241, Phe251, and Ile255 (Figure 3B); similar engagement is seen in the structure of IGF-I in complex with holo-IGF-1R (Li et al., 2019). The interaction between IGF-I and IR domain FnIII-1 is sparse, though potential salt bridges occur between IR domain FnIII-1 residue Arg539 and IGF-I residues Glu3 and Glu9 (Figure 3C). IR Arg539 is not conserved in IGF-1R, nor are IGF-I Glu3 or Glu9 conserved in human insulin, implying that these putative receptor-to-ligand salt bridges are unique to the IGF-I-to-IR interaction. However, IGF-I Glu3 and Glu9 are conserved in IGF-II, suggesting that equivalent salt bridges may occur in an [IGF-II]-bound hybrid receptor or in an [IGF-II]-bound IR.

The IR αCT segment

The IR αCT segment is observed here in a domain environment distinct from that seen in the homodimeric liganded structures of IR. However, the engagement of the IR αCT segment with IGF-I and with domain L1 of the IGF-1R monomer mimics that of the IGF-1R αCT segment within [IGF-I]-bound homodimeric IGF-1R (Figure 4A). Comparison shows that the residues of the respective αCT segments that engage IGF-I are conserved, whereas those of the IR αCT segment that engage the IGF-1R domain L1 display variation with respect to their counterparts in the [IGF-I]-bound IGF-1R homodimer. In particular, the IR αCT segment engages IGF-I via strictly conserved residues His710 (≡ IGF-1R His697) and Phe714 (≡ IGF-1R Phe701) but engages the IGF-1R domain L1 via less-strictly conserved residues Phe701 (≡ IGF-1R Tyr688), Phe705 (≡ IGF-1R Phe692), Tyr708 (≡ IGF-1R Phe695), Leu709 (≡ IGF-1R Leu696), Val712 (≡ IGF-1R Ser699), and Val713 (≡ IGF-1R Ile700). Despite the latter differences, the disposition observed here of the largely helical IR αCT segment on the surface of domain L1 is effectively identical to that of its counterpart in IGF-1R (Figure 4A).

The IR αCT segment also engages HybZip IR domain FnIII-1 and HybZip IGF-1R domain L2 (Figure 4B). Here, possible salt bridges occur between the set comprising IR αCT residues Lys703, Glu706, and Asp707 and the set comprising IR domain FnIII-1 residues Asp496 and Arg497—similar interactions between these residue sets are seen in the cryo-EM structures of insulin-complexed IR. IR αCT residue Glu697 has proximity to IGF-1R domain L2 residue Arg336, suggesting a possible single salt bridge between these components (Figure 4B), unique to the hybrid receptor as Arg336 in not conserved in IR.

The IGF-1R αCT segment

The IGF-1R αCT segment is located on the surface of the ligand-free IR domain L1 in the canonical ligand-free disposition of the αCT segment (i.e., with its axis directed at about 45° to the direction of the L1 domain β strands; Xu et al., 2018) (Figure 4C). As far as can be discerned, the segment engages IR domain L1 via the same residues (IGF-1R Tyr688, Phe792, and Phe795) that it does the cognate IGF-1R domain L1 within the apo IGF-1R ectodomain crystal structure (PDB: 5U8R; Xu et al., 2018) (Figure 4C). The C-terminal (non-helical) region of the IGF-1R αCT segment appears also to associate with the adjacent IGF-1R domain FnIII-2 in a fashion analogous to that seen in the apo IGF-1R ectodomain crystal structure (Xu et al., 2018). This association appears unique to IGF-1R—in the apo IR ectodomain, the C-terminal region of the αCT segment is not directed onto the adjacent FnIII-2 domain. We speculate that this difference may relate to the distinct topologies of the ligands (two-chain versus single-chain), with the apo αCT segment having to thread upon binding through the growth factor loop formed by its connecting C domain.

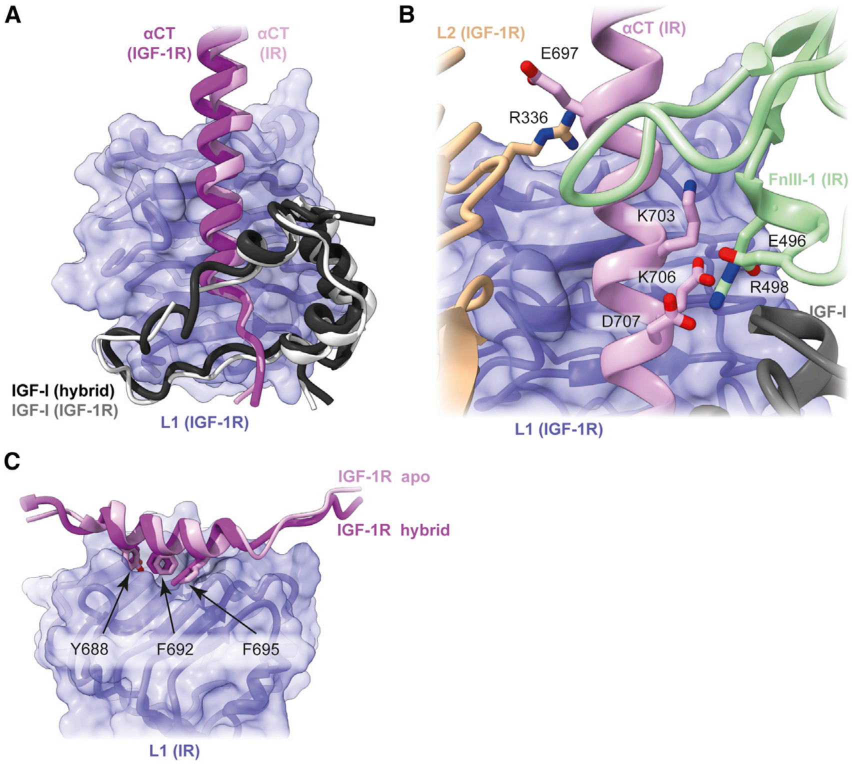

Figure 4. Arrangement of the αCT segments within [IGF-I]-complexed HybZip

(A) Schematic showing the close correspondence of the IR αCT segment on IGF-1R domain L1 within the context of [IGF-I]-bound HybZip with that of the IGF-1R αCT segment within the context of [IGF-I]-bound IGF-1R.

(B) Schematic showing putative salt bridges formed by the IR αCT segment with adjacent domains IGF-1R L2 and IR FnIII-1 within the context of [IGF-I]-bound HybZip.

(C) Schematic showing the close correspondence of the IGF-1R αCT segment on IR domain L1 within the context of [IGF-I]-bound HybZip with that of the IGF-1R αCT segment within the context of apo IGF-1R.

DISCUSSION

Tyrosine kinase receptors (TKRs) from diverse families, in particular the epidermal growth factor family, form heterogeneous multimers through non-covalent association of monomers. By contrast, IR and IGF-1R are homodimers stably connected by disulfide bonds (occurring during the final stage of cellular expression) and can also form committed heterodimeric receptors if both IR and IGF-1R monomers are expressed in the same cells. The structure we present here demonstrates that the heterodimeric class of receptors produces active, signaling receptors that bind to IGF-I in a similar way to that seen in homodimeric IGF-1R. Within these hybrids, IGF-I is seen bound to one side of the heterodimer (i.e., that which contains IGF-1R domains L1, CR, and L2 and the IR domains FnIII-1 and αCT) and to do so in a fashion analogous to that seen in [single-ligand]-complexed versions of the respective homodimeric receptors (Weis et al., 2018; Li et al., 2019; Xu et al., 2020). Our view of the activation of the homodimeric receptors upon ligand binding is anchored by the need for the respective intracellular tyrosine kinases to *trans*-autophosphorylate. The leucine-zipper constraint of HybZip is not sufficient to prevent a substantial dilation of the fibronectin domains; however, we see in our structure similar proximity of FnIII domains to those of liganded IR and IGF-1R, further supporting the thesis that the extracellular-domain rearrangements triggered by ligand binding bring about the close apposition of the tyrosine kinase domains.

Cryo-EM investigation has produced insulin-liganded forms of IR that are of higher stoichiometry than 1:1 (namely, with 2:1 and 4:1 insulin-to-receptor ratios), with the physiological relevance and interpretation of these forms being subject to debate, given that they are produced at supra-physiological high concentrations of insulin and, indeed, receptor. The 2:1 form has insulin complexed symmetrically to the two L1+αCT' primary binding sites, with each insulin interacting further with the adjacent domain FnIII-1' (where ' denotes elements from the alternate receptor monomer). We could envisage this 2:1 structure being sampled along with the 1:1 ratio structure, as local ligand and receptor concentration fluctuations may allow a small proportion to form. The 4:1 structures are similar but have a further two insulins bound to the respective domains FnIII-1 and FnIII-1' at sites distal to those that engage the insulins attached to the L1+αCT' elements. These latter two sites arguably reflect sites of transient insulin engagement that may affect the exposure of the L1+αCT' tandem element to insulin (Lawrence, 2021). However, no cryo-EM structure of liganded IGF-1R has emerged that displays a higher stoichiometric ratio than 1:1, and the same may ultimately prove to be the case for the hybrid receptor as well. We speculate that this difference likely reflects the different kinetic and/or thermodynamic properties of the respective homodimeric receptors. In particular, the isolated ectodomain of IGF-1R displays negative cooperativity of ligand binding (Surinya et al., 2008) whereas that of IR does not (Markussen et al., 1991). Furthermore, negative cooperativity of insulin binding to holo-IR is reduced at high insulin concentrations but is not for IGF-I binding to holo-IGF-1R—the reduced degree of negative cooperativity of IR at high insulin concentrations has been proposed to relate to formation of states of higher stoichiometric ratio (De Meyts and Whittaker, 2002).

We note that the structure presented here is the only one to date that is based on the B (exon 11+) isoform of human IR. IR-B has a 12-residue insert two residues upstream of the α-chain C terminus of the A (exon 11-) isoform (Seino and Bell, 1989), with the IR-B α-chain C-terminal residue thus being Ser731 as opposed to Ser719 in IR-A. Within our structure, the most C-terminally observed residue of the IR α chain is Lys718, i.e., we do not see evidence of the 12-residue extension. Nevertheless, the path of the α-chain C-terminal region is clear

CellPress
OPEN ACCESS

Table 1. Map and model building statistics for the IGF-I-complexed HybZip[a]

	Head	Legs
PDB code	7S0Q	7S8V
Composition (#)		
Chains	3	2
Atoms (including hydrogens)	15,551	12,651
Protein residues	958	782
Glycan residues	16	12
Bonds (RMSD)		
Length (Å) (# > 4σ)	0.003 (0)	0.003 (0)
Angles (°) (# > 4σ)	0.521 (0)	0.590 (0)
MolProbity score	2.07	1.74
Clash score	9.97	5.45
Ramachandran plot (%)		
Outliers/allowed/favored	0.00/9.92/90.08	0.00/7.03/92.97
Rotamer outliers (%)	0.00	0.14
C^{β} outliers (%)	0.00	0.00
Peptide plane (%)		
Cis proline/general	2.4/0.00	1.9/0.0
Twisted proline/general	0.0/0.0	0.0/0.1
C^{α}BLAM outliers (%)	4.80	3.05
ADP		
Iso/aniso (# atoms)	7,891/0	6,457/0
Protein (min/max/mean)	69.99/206.73/118.92	112.49/458.95/186.24
Glycan (min/max/mean)	93.63/190.15/135.24	124.02/182.08/158.68
Occupancy (# atoms)		
Occ = 1/0.5 /0.0	15,551/0/0	12,651/0/0
Map		
Resolution (Å): FSC independent half maps	3.70	3.73
Local resolution range (Å)	2.8–8.0	2.8–8.0
Sharpening *B* factor ($Å^2$)	-35.7	-45.7
Model versus map		
CC_{mask}	0.71	0.65
CC_{box}	0.82	0.68
CC_{volume}	0.71	0.65
CC individual chains		
HybZip IGF-1R/IR	0.77/0.72	0.68/0.70
IGF-I	0.73	N/A
Glycan IGF-1R/IR	0.68/0.71	0.58/0.62
Resolution (Å): FSC, masked map versus model at 0.143	3.64	3.80

RMSD, root-mean-square deviation; N/A, not applicable.
[a]See also Figures S3 and S4.

in our maps, confirming that it threads through the loop formed by the helical elements of IGF-I and its C domain. Significant entropic penalty would be associated with such threading, perhaps underscoring the fact that we do not yet have a complete understanding of the conformational pathway via which ligands bind to the IR/IGF-1R receptor family. A number of structural findings have very recently emerged (since the initial submission of this manuscript) that indicate that insulin binding to IR involves states wherein the insulin straddles sites 2' and 1 (Nielsen et al., 2022; Xiong et al., 2022). We speculate that such an event may, in the case of IGFs, be part of a process that reduces the energetic barrier to the ligand loop threading by the αCT segment, both in IGF-1R and in the hybrid receptor. We note also that within our model IGF-I C domain residues Arg36 and Arg37, although not laying in well-defined map density, lie in the vicinity of IGF-1R residues Glu303 and Glu304 within HybZip. It is thus also possible that interactions between these sets of residues facilitate loop opening (in the case of

IGF-I binding to IGF-1R, dual mutation of Arg36 and Arg37 to alanine result in a ca. 5-fold reduction in affinity [Jansson et al., 1998]).

Insulin has ca. two orders lower affinity for the hybrid receptor than IGF-I (Slaaby et al., 2006). The reasons for this reduced affinity are not immediately apparent from our structure. A variety of chimeric-receptor studies nevertheless provide clues as to the source of the reduced affinity for insulin. First, the insulin's site 1 within the hybrid likely comprises the IR L1-CR-L2 module and the IGF-1R αCT' element (as that combination has significantly higher affinity for insulin than the alternate combination [Kristensen et al., 1999]). However, its affinity for insulin is still less than that of the cognate IR L1-CR-L2 plus αCT pair. If insulin bound to the IR L1-CR-L2 module and the IGF-1R αCT' element, then the insulin's site 2 would reside on the IGF-1R domain FnIII-1' within the hybrid. Substitution within the hybrid receptor of at least part of IGF-1R domain FnIII-1 with its IR counterpart increases insulin affinity by an order of magnitude (Benyoucef et al., 2007). It is thus possible that at least part of the reduced affinity of the hybrid receptor for insulin is a consequence of the reduced affinity of insulin for site 2 on IGF-1R. Further structural investigation of these issues will require structures of the hybrid receptor in complex with multiple insulin molecules.

The structure of an apo hybrid receptor is not yet available. Determination of the structure of apo IR and IGF-1R has thus far proved possible only via X-ray crystallography (McKern et al., 2006; Smith et al., 2010; Croll et al., 2016; Xu et al., 2018), with high-resolution cryo-EM investigation being hampered presumably by the conformational variability of the receptor ectodomain in the absence of membrane anchoring (Scapin et al., 2018; Gutmann et al., 2020). Nevertheless, the structure presented here contributes a key piece to the emerging repertoire of liganded structures from this receptor family and allows discernment of both common and divergent elements. We now possess a suite of structures describing the interactions of insulin and IGFs to IR, IGF-1R, and a hybrid receptor of IR and IGF-1R as well as apo receptor structures of IR and IGF-1R and which describe a plethora of conformational subtlety.

STAR★METHODS

Detailed methods are provided in the online version of this paper and include the following:

- KEY RESOURCES TABLE
- RESOURCE AVAILABILITY
  - Lead contact
  - Materials availability
  - Data and code availability
- EXPERIMENTAL MODEL AND SUBJECT DETAILS
  - CHO Lec8 cells
  - IGF-IR null fibroblasts overexpressing hIR-B
  - BALB/c3T3 cells (P6) overexpressing IGF-IR
- METHOD DETAILS
  - HybZip cloning and production
  - Preparation of mAb 18-44 affinity column
  - Purification of HybZip
  - Competition receptor binding assay
  - CryoEM data collection
  - Three-dimensional reconstruction
  - Model generation and real-space refinement
- QUANTIFICATION AND STATISTICAL ANALYSIS

SUPPLEMENTAL INFORMATION

Supplemental information can be found online at https://doi.org/10.1016/j.str.2022.05.007.

ACKNOWLEDGMENTS

M.C.L. acknowledges financial support from the Australian National Health and Medical Research Council (NHMRC) Project Grant APP1128553; his institute receives Victorian State Government Operational Infrastructure Support and funding from the Australian NHMRC Independent Research Institutes Infrastructure Support Scheme. The authors acknowledge the use of instruments and assistance at the Monash Ramaciotti Centre for Cryo-Electron Microscopy, a Node of Microscopy Australia. This research used equipment funded by Australian Research Council grant LE120100090. T.I.C. is supported by the Wellcome Trust Principal Research Fellowship of Prof. Randy Read (grant number 209407/Z/17/Z). This research was funded in whole, or in part, by the Wellcome Trust grant number 209407/Z/17/Z. For the purpose of open access, the author has applied a CC BY public copyright license to any author-accepted manuscript version arising from this submission. The authors thank Prof. Ken Siddle (University of Cambridge, UK) for providing the hybridomas expressing mAb 18-44 and Dr. Oliver Clarke (Columbia University, USA) for advice regarding cryo-EM software.

AUTHOR CONTRIBUTIONS

M.B.M. performed molecular and cell biology. Y.X. and M.B.M. performed protein purification and sample preparation. J.G.M. developed the 18-44 resin pull-down technology. H.V. and Y.X. performed cryo-EM experiments. C.D. performed assays. Y.X. performed cryo-EM 3D reconstruction. T.I.C., Y.X., and M.C.L. performed model building and refinement. N.S.K. contributed cryo-EM expertise. M.C.L. designed the project. M.C.L. and B.E.F. analyzed results and wrote the manuscript. All authors read and commented on the final manuscript.

DECLARATION OF INTERESTS

M.C.L.'s laboratory has a funded agreement with Eli Lilly and Company to conduct research not connected to this publication.

Received: December 7, 2021
Revised: April 11, 2022
Accepted: May 10, 2022
Published: August 4, 2022

REFERENCES

Afonine, P.V., Poon, B.K., Read, R.J., Sobolev, O.V., Terwilliger, T.C., Urzhumtsev, A., and Adams, P.D. (2018). Real-space refinement in PHENIX for cryo-EM and crystallography. Acta Crystallogr. D. Biol. Crystallogr. *74*, 531–544. https://doi.org/10.1107/S2059798318006551.

Arnold, S.E., Arvanitakis, Z., Macauley-Rambach, S.L., Koenig, A.M., Wang, H.Y., Ahima, R.S., Craft, S., Gandy, S., Buettner, C., Stoeckel, L.E., et al. (2018). Brain insulin resistance in type 2 diabetes and Alzheimer disease: concepts and conundrums. Nat. Rev. Neurol. *14*, 168–181. https://doi.org/10.1038/nrneurol.2017.185.

Bailyes, E.M., Nave, B.T., Soos, M.A., Orr, S.R., Hayward, A.C., and Siddle, K. (1997). Insulin receptor/IGF-I receptor hybrids are widely distributed in mammalian tissues: quantification of individual receptor species by selective immunoprecipitation and immunoblotting. Biochem. J. *327*, 209–215. https://doi.org/10.1042/bj3270209.

Belfiore, A. (2007). The role of insulin receptor isoforms and hybrid insulin/IGF-I receptors in human cancer. Curr. Pharm. Des. *13*, 671–686. https://doi.org/10.2174/138161207780249173.

Belfiore, A., Malaguarnera, R., Vella, V., Lawrence, M.C., Sciacca, L., Frasca, F., Morrione, A., and Vigneri, R. (2017). Insulin receptor isoforms in physiology and disease: an updated view. Endocr. Rev. *38*, 379–431. https://doi.org/10.1210/er.2017-00073.

Benyoucef, S., Surinya, K.H., Hadaschik, D., and Siddle, K. (2007). Characterization of insulin/IGF hybrid receptors: contributions of the insulin receptor L2 and Fn1 domains and the alternatively spliced exon 11 sequence to ligand binding and receptor activation. Biochem. J. *403*, 603–613. https://doi.org/10.1042/bj20061709.

Chitnis, M.M., Yuen, J.S., Protheroe, A.S., Pollak, M., and Macaulay, V.M. (2008). The type 1 insulin-like growth factor receptor pathway. Clin. Cancer Res. *14*, 6364–6370. https://doi.org/10.1158/1078-0432.ccr-07-4879.

Croll, T.I. (2018). ISOLDE: a physically realistic environment for model building into low-resolution electron-density maps. Acta Crystallogr. D. Biol. Crystallogr. *74*, 519–530. https://doi.org/10.1107/s2059798318002425.

Croll, T.I., and Read, R.J. (2021). Adaptive Cartesian and torsional restraints for interactive model rebuilding. Acta Crystallogr. D Struct. Biol *77*, 438–446. https://doi.org/10.1107/s2059798321001145.

Croll, T.I., Smith, B.J., Margetts, M.B., Whittaker, J., Weiss, M.A., Ward, C.W., and Lawrence, M.C. (2016). Higher-resolution structure of the human insulin receptor ectodomain: multi-modal inclusion of the insert domain. Structure *24*, 469–476. https://doi.org/10.1016/j.str.2015.12.014.

De Meyts, P., and Whittaker, J. (2002). Structural biology of insulin and IGF1 receptors: implications for drug design. Nat. Rev. Drug Discov. *1*, 769–783. https://doi.org/10.1038/nrd917.

Denley, A., Bonython, E.R., Booker, G.W., Cosgrove, L.J., Forbes, B.E., Ward, C.W., and Wallace, J.C. (2004). Structural determinants for high-affinity binding of insulin-like growth factor II to insulin receptor (IR)-A, the exon 11 minus isoform of the IR. Mol. Endocrinol. *18*, 2502–2512. https://doi.org/10.1210/me.2004-0183.

Emsley, P., Lohkamp, B., Scott, W.G., and Cowtan, K. (2010). Features and development of Coot. Acta Crystallogr. D. Biol. Crystallogr. *66*, 486–501. https://doi.org/10.1107/S0907444910007493.

Goddard, T.D., Huang, C.C., Meng, E.C., Pettersen, E.F., Couch, G.S., Morris, J.H., and Ferrin, T.E. (2018). UCSF ChimeraX: meeting modern challenges in visualization and analysis. Protein Sci. *27*, 14–25. https://doi.org/10.1002/pro.3235.

Gutmann, T., Kim, K.H., Grzybek, M., Walz, T., and Coskun, Ü. (2018). Visualization of ligand-induced transmembrane signaling in the full-length human insulin receptor. J. Cell Biol. *217*, 1643–1649. https://doi.org/10.1083/jcb.201711047.

Gutmann, T., Schäfer, I.B., Poojari, C., Brankatschk, B., Vattulainen, I., Strauss, M., and Coskun, Ü. (2020). Cryo-EM structure of the complete and ligand-saturated insulin receptor ectodomain. J. Cell Biol. *219*, e201907210. https://doi.org/10.1083/jcb.201907210.

Hilpert, K., Hansen, G., Wessner, H., Kuttner, G., Welfle, K., Seifert, M., and Hohne, W. (2001). Anti-c-myc antibody 9E10: epitope key positions and variability characterized using peptide spot synthesis on cellulose. Protein Eng. *14*, 803–806. https://doi.org/10.1093/protein/14.10.803.

Hoyne, P.A., Cosgrove, L.J., McKern, N.M., Bentley, J.D., Ivancic, N., Elleman, T.C., and Ward, C.W. (2000). High affinity insulin binding by soluble insulin receptor extracellular domain fused to a leucine zipper. FEBS Lett. *479*, 15–18. https://doi.org/10.1016/s0014-5793(00)01872-x.

Jansson, M., Andersson, G., Uhlén, M., Nilsson, B., and Kördel, J. (1998). The insulin-like growth factor (IGF)binding protein 1 binding epitope on IGF-I probed by heteronuclear NMR spectroscopy and mutational analysis. J. Biol. Chem. *273*, 24701–24707. https://doi.org/10.1074/jbc.273.38.24701.

King, R., Wells, J.R.E., Krieg, P., Snoswell, M., Brazier, J., Bagley, C.J., Wallace, J.C., Ballard, F.J., Ross, M., and Francis, G.L. (1992). Production and characterization of recombinant insulin-like growth factor-I (IGF-I) and potent analogues of IGF-I, with Gly or Arg substituted for Glu3, following their expression in Escherichia coli as fusion proteins. J. Mol. Endocrinol. *8*, 29–41. https://doi.org/10.1677/jme.0.0080029.

Kristensen, C., Wiberg, F.C., and Andersen, A.S. (1999). Specificity of insulin and insulin-like growth factor I receptors investigated using chimeric mini-receptors. J. Biol. Chem. *274*, 37351–37356. https://doi.org/10.1074/jbc.274.52.37351.

Lawrence, C.F., Margetts, M.B., Menting, J.G., Smith, N.A., Smith, B.J., Ward, C.W., and Lawrence, M.C. (2016). Insulin mimetic peptide disrupts the primary binding site of the insulin receptor. J. Biol. Chem. *291*, 15473–15481. https://doi.org/10.1074/jbc.m116.732180.

Lawrence, M.C. (2021). Understanding insulin and its receptor from their three-dimensional structure. Mol. Metabol. 101255. https://doi.org/10.1016/j.molmet.2021.101255.

Li, J., Choi, E., Yu, H., and Bai, X.C. (2019). Structural basis of the activation of type 1 insulin-like growth factor receptor. Nat. Commun. *10*, 4567. https://doi.org/10.1038/s41467-019-12564-0.

Lou, M., Garrett, T.P.J., McKern, N.M., Hoyne, P.A., Epa, V.C., Bentley, J.D., Lovrecz, G.O., Cosgrove, L.J., Frenkel, M.J., and Ward, C.W. (2006). The first three domains of the insulin receptor differ structurally from the insulin-like growth factor 1 receptor in the regions governing ligand specificity. Proc. Natl. Acad. Sci. U S A *103*, 12429–12434. https://doi.org/10.1073/pnas.0605395103.

Markussen, J., Halstrøm, J., Wiberg, F.C., and Schäffer, L. (1991). Immobilized insulin for high capacity affinity chromatography of insulin receptors. J. Biol. Chem. *266*, 18814–18818. https://doi.org/10.1016/s0021-9258(18)55136-x.

McKern, N.M., Lawrence, M.C., Streltsov, V.A., Lou, M.Z., Adams, T.E., Lovrecz, G.O., Elleman, T.C., Richards, K.M., Bentley, J.D., Pilling, P.A., et al. (2006). Structure of the insulin receptor ectodomain reveals a folded-over conformation. Nature *443*, 218–221. https://doi.org/10.1038/nature05106.

McKern, N.M., Lou, M., Frenkel, M.J., Verkuylen, A., Bentley, J.D., Lovrecz, G.O., Ivancic, N., Elleman, T.C., Garrett, T.P., Garrett, T.P.J., et al. (1997). Crystallization of the first three domains of the human insulin-like growth factor-1 receptor. Protein Sci. *6*, 2663–2666. https://doi.org/10.1002/pro.5560061223.

Menting, J.G., Lawrence, C.F., Kong, G.K., Margetts, M.B., Ward, C.W., and Lawrence, M.C. (2015). Structural congruency of ligand binding to the insulin and insulin/type 1 insulin-like growth factor hybrid receptors. Structure *23*, 1271–1282. https://doi.org/10.1016/j.str.2015.04.016.

Menting, J.G., Whittaker, J., Margetts, M.B., Whittaker, L.J., Kong, G.K.W., Smith, B.J., Watson, C.J., Žáková, L., Kletvíková, E., Jiráček, J., et al. (2013). How insulin engages its primary binding site on the insulin receptor. Nature *493*, 241–245. https://doi.org/10.1038/nature11781.

Menting, J.G., Yang, Y., Chan, S.J., Phillips, N.B., Smith, B.J., Whittaker, J., Wickramasinghe, N.P., Whittaker, L.J., Pandyarajan, V., Wan, Z.L., et al. (2014). Protective hinge in insulin opens to enable its receptor engagement. Proc. Natl. Acad. Sci. U S A *111*, E3395–E3404. https://doi.org/10.1073/pnas.1412897111.

Moxham, C.P., Duronio, V., and Jacobs, S. (1989). Insulin-like growth factor I receptor beta-subunit heterogeneity. Evidence for hybrid tetramers composed of insulin-like growth factor I and insulin receptor heterodimers. J. Biol. Chem. *264*, 13238–13244. https://doi.org/10.1016/S0021-9258(18)51620-3.

Mughal, R.S., Bridge, K., Buza, I., Slaaby, R., Worm, J., Klitgaard-Povlsen, G., Hvid, H., Schiødt, M., Cubbon, R., Yuldasheva, N., et al. (2018). Effects of obesity on insulin: insulin-like growth factor 1 hybrid receptor expression and Akt phosphorylation in conduit and resistance arteries. Diab. Vasc. Dis. Res. *16*, 160–170. https://doi.org/10.1177/1479164118802550.

Nakane, T., Kimanius, D., Lindahl, E., and Scheres, S.H. (2018). Characterisation of molecular motions in cryo-EM single-particle data by multi-body refinement in RELION. Elife *7*, e36861. https://doi.org/10.7554/elife.36861.

Nielsen, J., Brandt, J., Boesen, T., Hummelshøj, T., Slaaby, R., Schluckebier, G., and Nissen, P. (2022). Structural investigations of full-length insulin receptor dynamics and signalling. J. Mol. Biol. *434*, 167458. https://doi.org/10.1016/j.jmb.2022.167458.

O'Shea, E.K., Klemm, J.D., Kim, P.S., and Alber, T. (1991). X-ray structure of the GCN4 leucine zipper, a two-stranded, parallel coiled coil. Science *254*, 539–544. https://doi.org/10.1126/science.1948029.

Pandini, G., Frasca, F., Mineo, R., Sciacca, L., Vigneri, R., and Belfiore, A. (2002). Insulin/insulin-like growth factor I hybrid receptors have different

biological characteristics depending on the insulin receptor isoform involved. J. Biol. Chem. *277*, 39684–39695. https://doi.org/10.1074/jbc.m202766200.

Pettersen, E.F., Goddard, T.D., Huang, C.C., Couch, G.S., Greenblatt, D.M., Meng, E.C., and Ferrin, T.E. (2004). UCSF Chimera—a visualization system for exploratory research and analysis. J. Comput. Chem. *25*, 1605–1612. https://doi.org/10.1002/jcc.20084.

Pietrzkowski, Z., Lammers, R., Carpenter, G., Soderquist, A.M., Limardo, M., Phillips, P.D., Ullrich, A., and Baserga, R. (1992). Constitutive expression of insulin-like growth factor 1 and insulin-like growth factor 1 receptor abrogates all requirements for exogenous growth factors. Cell Growth Differ. *3*, 199–205.

Prigent, S.A., Stanley, K.K., and Siddle, K. (1990). Identification of epitopes on the human insulin receptor reacting with rabbit polyclonal antisera and mouse monoclonal antibodies. J. Biol. Chem. *265*, 9970–9977. https://doi.org/10.1016/s0021-9258(19)38765-4.

Punjani, A., Rubinstein, J.L., Fleet, D.J., and Brubaker, M.A. (2017). cryoSPARC: algorithms for rapid unsupervised cryo-EM structure determination. Nat. Methods *14*, 290–296. https://doi.org/10.1038/nmeth.4169.

Saltiel, A.R., and Kahn, C.R. (2001). Insulin signalling and the regulation of glucose and lipid metabolism. Nature *414*, 799–806. https://doi.org/10.1038/414799a.

Scapin, G., Dandey, V.P., Zhang, Z., Prosise, W., Hruza, A., Kelly, T., Mayhood, T., Strickland, C., Potter, C.S., and Carragher, B. (2018). Structure of the insulin receptor–insulin complex by single-particle cryo-EM analysis. Nature *556*, 122–125. https://doi.org/10.1038/nature26153.

Schumacher, R., Mosthaf, L., Schlessinger, J., Brandenburg, D., and Ullrich, A. (1991). Insulin and insulin-like growth factor-1 binding specificity is determined by distinct regions of their cognate receptors. J. Biol. Chem. *266*, 19288–19295. https://doi.org/10.1016/s0021-9258(18)54996-6.

Seino, S., and Bell, G.I. (1989). Alternative splicing of human insulin receptor messenger RNA. Biochem. Biophys. Res. Commun. *159*, 312–316. https://doi.org/10.1016/0006-291x(89)92439-x.

Slaaby, R., Schäffer, L., Lautrup-Larsen, I., Andersen, A.S., Shaw, A.C., Mathiasen, I.S., and Brandt, J. (2006). Hybrid receptors formed by insulin receptor (IR) and insulin-like growth factor I receptor (IGF-IR) have low insulin and high IGF-1 affinity irrespective of the IR splice variant. J. Biol. Chem. *281*, 25869–25874. https://doi.org/10.1074/jbc.m605189200.

Smith, B.J., Huang, K., Kong, G., Chan, S.J., Nakagawa, S., Menting, J.G., Hu, S.-Q., Whittaker, J., Steiner, D.F., Katsoyannis, P.G., et al. (2010). Structural resolution of a tandem hormone-binding element in the insulin receptor and its implications for design of peptide agonists. Proc. Natl. Acad. Sci. U S A *107*, 6771–6776. https://doi.org/10.1073/pnas.1001813107.

Soos, M.A., and Siddle, K. (1989). Immunological relationships between receptors for insulin and insulin-like growth factor I. Evidence for structural heterogeneity of insulin-like growth factor I receptors involving hybrids with insulin receptors. Biochem. J. *263*, 553–563. https://doi.org/10.1042/bj2630553.

Soos, M.A., Field, C.E., Lammers, R., Ullrich, A., Zhang, B., Roth, R.A., Andersen, A.S., Kjeldsen, T., and Siddle, K. (1992). A panel of monoclonal antibodies for the type I insulin-like growth factor receptor. Epitope mapping, effects on ligand binding, and biological activity. J. Biol. Chem. *267*, 12955–12963. https://doi.org/10.1016/s0021-9258(18)42367-8.

Soos, M.A., Siddle, K., Baron, M.D., Heward, J.M., Luzio, J.P., Bellatin, J., and Lennox, E.S. (1986). Monoclonal antibodies reacting with multiple epitopes on the human insulin receptor. Biochem. J. *235*, 199–208. https://doi.org/10.1042/bj2350199.

Soos, M.A., Whittaker, J., Lammers, R., Ullrich, A., and Siddle, K. (1990). Receptors for insulin and insulin-like growth factor-I can form hybrid dimers. Characterisation of hybrid receptors in transfected cells. Biochem. J. *270*, 383–390. https://doi.org/10.1042/bj2700383.

Surinya, K.H., Forbes, B.E., Occhiodoro, F., Booker, G.W., Francis, G.L., Siddle, K., Wallace, J.C., and Cosgrove, L.J. (2008). An investigation of the ligand binding properties and negative cooperativity of soluble insulin-like growth factor receptors. J. Biol. Chem. *283*, 5355–5363. https://doi.org/10.1074/jbc.m707054200.

Taniguchi, C.M., Emanuelli, B., and Kahn, C.R. (2006). Critical nodes in signalling pathways: insights into insulin action. Nat. Rev. Mol. Cell Biol. *7*, 85–96. https://doi.org/10.1038/nrm1837.

Titone, R., Zhu, M., and Robertson, D.M. (2018). Insulin mediates de novo nuclear accumulation of the IGF-1/insulin Hybrid Receptor in corneal epithelial cells. Sci. Rep. *8*, 4378. https://doi.org/10.1038/s41598-018-21031-7.

Uchikawa, E., Choi, E., Shang, G., Yu, H., and Bai, X.-c. (2019). Activation mechanism of the insulin receptor revealed by cryo-EM structure of the fully liganded receptor-ligand complex. Elife *8*, e48630. https://doi.org/10.7554/eLife.48630.

Ullrich, A., Gray, A., Tam, A.W., Yang-Feng, T., Tsubokawa, M., Collins, C., Henzel, W., Le Bon, T., Kathuria, S., Chen, E., et al. (1986). Insulin-like growth factor I receptor primary structure: comparison with insulin receptor suggests structural determinants that define functional specificity. EMBO J. *5*, 2503–2512. https://doi.org/10.1002/j.1460-2075.1986.tb04528.x.

Weis, F., Menting, J.G., Margetts, M.B., Chan, S.J., Xu, Y., Tennagels, N., Wohlfart, P., Langer, T., Müller, C.W., Dreyer, M.K., and Lawrence, M.C. (2018). The signalling conformation of the insulin receptor ectodomain. Nat. Commun. *9*, 4420. https://doi.org/10.1038/s41467-018-06826-6.

Xiong, X., Blakely, A., Kim, J.H., Menting, J.G., Schafer, I.B., Schubert, H.L., Agrawal, R., Gutmann, T., Delaine, C., Zhang, Y.W., et al. (2022). Symmetric and asymmetric receptor conformation continuum induced by a new insulin. Nat. Chem. Biol. *18*, 511–519. https://doi.org/10.1038/s41589-022-00981-0.

Xiong, X., Menting, J.G., Disotuar, M.M., Smith, N.A., Delaine, C.A., Ghabash, G., Agrawal, R., Wang, X., He, X., Fisher, S.J., et al. (2020). A structurally minimized yet fully active insulin based on cone-snail venom insulin principles. Nat. Struct. Mol. Biol. *27*, 615–624. https://doi.org/10.1038/s41594-020-0430-8.

Xu, Y., Kirk, N.S., Venugopal, H., Margetts, M.B., Croll, T.I., Sandow, J.J., Webb, A.I., Delaine, C.A., Forbes, B.E., and Lawrence, M.C. (2020). How IGF-II binds to the human type 1 insulin-like growth factor receptor. Structure *28*, 786–798.e6. https://doi.org/10.1016/j.str.2020.05.002.

Xu, Y., Kong, G.K.W., Menting, J.G., Margetts, M.B., Delaine, C.A., Jenkin, L.M., Kiselyov, V.V., De Meyts, P., Forbes, B.E., and Lawrence, M.C. (2018). How ligand binds to the type 1 insulin-like growth factor receptor. Nat. Commun. *9*, 821. https://doi.org/10.1038/s41467-018-03219-7.

Zhang, B., and Roth, R.A. (1991). A region of the insulin receptor important for ligand binding (residues 450-601) is recognized by patients' autoimmune antibodies and inhibitory monoclonal antibodies. Proc. Natl. Acad. Sci. U S A *88*, 9858–9862. https://doi.org/10.1073/pnas.88.21.9858.

Zhang, X., Yu, D., Sun, J., Wu, Y., Gong, J., Li, X., Liu, L., Liu, S., Liu, J., Wu, Y., et al. (2020). Visualization of ligand-bound ectodomain assembly in the full-length human IGF-1 receptor by cryo-EM single-particle analysis. Structure *28*, 555–561.e4. https://doi.org/10.1016/j.str.2020.03.007.

STAR★METHODS

KEY RESOURCES TABLE

REAGENT or RESOURCE	SOURCE	IDENTIFIER
Antibodies		
Mouse monoclonal antibody 24-60 (anti-human IGF-1R)	(Soos et al., 1992)	N/A
Mouse monoclonal antibody 83-7 (anti-human IR)	(Soos et al., 1986)	N/A
Mouse monoclonal antibody 18-44 (anti-human IR)	(Soos et al., 1986)	N/A
Mouse monoclonal antibody 9E10 (anti-c-myc)	CSIRO Laboratories, Parkville, Australia	ATCC CRL1729
IRDye 800CW Goat anti-mouse IgG	LI-COR Biosciences	Cat# 926-32210; RRID: AB_621842
Chemicals, peptides, and recombinant proteins		
9E10 c-myc epitope peptide, sequence EQKLISEEDL (>75% purity)	Genscript	N/A
18-44 IR epitope peptide, sequence TSPEEHRPFE (>70% purity)	Genscript	N/A
Receptor grade IGF-I	GroPep	Cat# CM001
IGF-I	(King et al., 1992)	N/A
Eu-IGF-I	(Denley et al., 2004)	N/A
Insulin	Novo Nordisk	Actrapid
Fetal calf serum	Scientifix	FBSFR-62147A
G418	ThermoFisher Scientific	10131035
Puromycin	ThermoFisher Scientific	A1113802
L-Methionine sulfoximine	Merck	GSS-1015-F
Eu-N1-ITC chelate	Perkin Elmer	1244-302
DELFIA Enhancement Solution	Perkin Elmer	1244-104
Fetal Bovine Serum, dialyzed	ThermoFisher Scientific	30067344
DMEM with Glucose, without L-Glutamine	Lonza	12-614F
GS Supplement	Merck	GSS-1016-C
Trypsin Gold	Promega	Cat# V5280
FuGENE HD transfection agent	Promega	E2311
ProSep-vA resin	Millipore	
Deposited data		
Crystal structure of apo IGF-1R ectodomain	(Xu et al., 2018)	PDB: 5U8R
Crystal structure of apo IR ectodomain	(Croll et al., 2016)	PDB: 4ZXB
CryoEM structure of IGF-II bound IGF-1R (head region; open conformation	(Xu et al., 2020)	PDB: 6VXG
Model: IGF-I-bound HybZip, head region	This study	PDB: 7S0Q
Model: IGF-I-bound HybZip, leg region	This study	PDB: 7S8V
Map: IGF-I-bound HybZip, head region	This study	EMD-24791
Map: IGF-I-bound HybZip, leg region	This study	EMD-24927
Experimental models: Cell lines		
BALB/c3T3 cells overexpressing IGF-IR	(Pietrzkowski et al., 1992)	P6
IGF-IR null mouse fibroblasts overexpressing the human IR-B	In-house; (Denley et al., 2004)	R$^-$IR-B
CHO-Lec8	ATCC	ATCC CRL-1737
Recombinant DNA		

(*Continued on next page*)

Continued		
REAGENT or RESOURCE	SOURCE	IDENTIFIER
IGF-1Rzip	(Xu et al., 2020)	N/A
IR-Bzip, custom synthesis and cloning	Genscript	N/A
pEE14 vector	Lonza	N/A
pJ509 vector	DNA 2.0	N/A
oligo 5'-GCGCGTCGACGCCT CCTTCAAGTCCCAG-3'	Integrated DNA Technologies	N/A
oligo 5'-GCGCTCTAGATTATT AATTCAGATCCTC-3'	Integrated DNA Technologies	N/A
**Software and algorithms**		
RELION v3.0.5	(Nakane et al., 2018)	https://www3.mrc-lmb.cam.ac.uk/relion
CryoSPARC v2.11	(Punjani et al., 2017)	https://cryosparc.com/
ISOLDE v1.03b	(Croll, 2018)	https://isolde.cimr.cam.ac.uk/
Phenix v1.16-3549-000	(Afonine et al., 2018)	https://www.phenix-online.org/
Coot v0.8.9.1	(Emsley et al., 2010)	https://www2.mrc-lmb.cam.ac.uk/personal/pemsley/coot/
Chimera v1.11.2	(Pettersen et al., 2004)	https://www.cgl.ucsf.edu/chimera/
ChimeraX v0.91	(Goddard et al., 2018)	https://www.cgl.ucsf.edu/chimerax/
Graphpad Prism v9.0.0	Graphpad Software	https://www.graphpad.com/scientific-software/prism/
**Other**		
Mini-Leak low divinylsulphone-activated resin	Kem-en-Tec	Cat# 1011 H
Sepharose CL-4B resin	GE Healthcare Lifesciences	Cat# 17015001
Sephadex-G75	GE Healthcare / Cytiva	Cat# 17005001
Pellicon 3 0.11 m^2 10 kDa Ultracel concentrator	Merck-Millipore	Cat# P3C010C01
Superdex 200 Increase 10/300 GL	GE Healthcare Lifesciences	Cat# 28990944
0.5 mL 10 kDa Amicon Ultra concentrator	Sigma-Aldrich	Cat# UFC501008
UltrAuFoil R1.2/1.3 300-mesh grids	Quantifoil	N/A
Pelco easiGlow	Ted Pella	Cat# 91000S-230
Vitrobot mark IV	ThermoFisher Scientific	N/A

RESOURCE AVAILABILITY

Lead contact

Further information and reasonable requests for resources and reagents should be directed to and will be fulfilled by the Lead Contact, Michael Lawrence (lawrence@wehi.edu.au).

Materials availability

There are restrictions to the availability of the vector and stable cell lines associated with the IGF-1Rzip construct due to the pEE14 vector being subject to a Research Agreement with Lonza.

Data and code availability

- The "head" and "leg" maps and their associated atomic models have been deposited in the Electron Microscopy Data Bank and Protein Data Bank (EMDB entries EMD-24791 and EMD-24927, and PDB entries 7S0Q and 7S8V, respectively).
- The paper does not report code.
- Any additional information required to re-analyze the data reported in this paper is available from the lead contact upon reasonable request.

EXPERIMENTAL MODEL AND SUBJECT DETAILS

CHO Lec8 cells

CH0 Lec8 cells (CRL1737-ATCC) stably transfected with IGF-1Rzip and IR-Bzip were cultured in a 5% CO_2 atmosphere at 37°C in DMEM (high glucose) media containing 25 mM methionine sulfoxide, 10 mg.mL^{-1} puromycin, 1× GS supplement and 10% dialysed fetal bovine serum. The sex of the cells is unknown. The cells are to the best of our knowledge authenticated.

IGF-IR null fibroblasts overexpressing hIR-B

IGF-IR null mouse fibroblasts overexpressing the human insulin receptor isoform B (Denley et al., 2004) were cultured in DMEM, 10% fetal calf serum, 1% penicillin/streptomycin, G418 (0.5 mM), at 37°C, 5% CO_2. The cells were validated for over-expression of IR-B by both PCR and FACS analysis. The sex of the cells is unknown.

BALB/c3T3 cells (P6) overexpressing IGF-IR

BALB/c3T3 overexpressing IGF-IR (P6) cells were cultured in DMEM, 10% fetal calf serum, 1% penicillin/streptomycin, G418 (0.5 mM), at 37°C, 5% CO_2. The P6 cells were a gift from Dr Renato Baserga (Pietrzkowski et al., 1992) and were validated for over-expression of IGF-1R by FACS analysis (Denley et al., 2004).

METHOD DETAILS

HybZip cloning and production

cDNA encoding residues 1–928 of IR-B followed at its C terminus by the 33-residue GCN4 zipper sequence RMKQLEDKVEE LLSKNYHLENEVARLKKLVGER (O'Shea et al., 1991) (Figure S1A) was synthesised by GenScript (USA) and then cloned by GenScript (USA) into the Hind III / EcoR1 sites of the pEE14 mammalian expression vector (Lonza) for stable expression of the protein ("IR-Bzip") in CHO Lec8 cells. Cells were transfected with complexes of plasmid DNA using FuGENE HD transfection agent (Promega, Australia) and cells were exposed to 25 μM methionine sulphoximine in Lonza DMEM (High Glucose) media containing 1× GS supplement (Merck) and 10% dialysed FBS (Life Technologies). Cells were plated in 96-well plates using limiting dilution and colonies allowed to form over several weeks. Secretion of IR-Bzip from colonies was detected via Western Blots using mAb 83-7 (Soos et al., 1986) (hybridomas expressing mAb 83-7 were a gift of Prof. Ken Siddle; Cambridge, UK). Dozens of colonies were amplified into twelve-well trays and later into tissue-culture flasks and monitored for expression. Several of the best-expressing clones were then further screened by seeding cells at exactly the same densities in six-well trays and individually monitored for expression over time. The single best-expressing clone was then selected for transfection with IGF-1Rzip to make a dual expressing cell line. The IGF-1Rzip synthetic gene (comprising residues 1-905 of IGF-1R followed by the 33-residue GCN4 zipper sequence, a three-serine spacer and the c-myc tag sequence EQKLISEEDLN (Xu et al., 2020) (Figure S1B)) was amplified using forward primer 5'-GCGCGTCGACGCCTCCTTCAAGTCCCAG-3' and reverse primer 5'-GCGCTCTAGATTATTAATTCAGATCCTC-3' and cloned into the Sal1/Xba1 sites of PiggyBac expression cassette pJ509 (DNA 2.0). Plasmid DNA containing the IGF-1Rzip gene was transfected into cells as described above, with the additional selection pressure of puromycin in the medium. Secretion of both IR-Bzip and IGF-1Rzip targets was monitored by Western blotting culture supernatants (Figure S2A) with mAb 83-7 and mAb 24-60, respectively (the latter also a gift from Prof. Ken Siddle, Cambridge, UK). Clones were chosen for scale-up based on selection of cell lines demonstrating qualitatively similar levels of secretion of both targets. The single best-expressing cell line was then selected to enter roller-bottle scale-up. Cells were seeded in roller bottles and allowed to grow for 10 days. At this point, 2.5 mM valproic acid (Sigma) was added and the cells were allowed to incubate for a further 4 days, at which stage the conditioned medium was decanted from the roller bottles and filtered for subsequent purification of receptor protein.

Preparation of mAb 18-44 affinity column

In the following sequence, 13 mL (settled volume) ProSep-vA resin (Millipore) was first washed with 2 bed volumes (BV) TBSA (25 mM Tris-HCl, 137 mM NaCl, 2.7 mM KCl, pH 8.0 + 0.02 % NaN_3), cleaned with 0.2 M glycine-HCl, pH 2.5 (2 BV), washed with TBSA (2 BV), pH was adjusted with 10 mL 3 M Tris-HCl (pH 8.5) and then equilibrated with 10 BV of TBSA.

Seven 20 mL aliquots of frozen mAb 18-44 hybridoma culture supernatant were obtained as a gift from Prof. Kenneth Siddle (University of Cambridge, UK); these were then thawed rapidly by agitation in warm water and combined. NaN_3 was added to 0.05 % (w/v) as well as 5 mL per litre 3 M Tris-HCl, pH 8.5. The combined samples were filtered through a 0.2 μm bottle filter (Thermo Fisher Scientific) immediately before use. The entire sample was run under gravity through the above resin and the column was washed with ~100 mL TBSA. Bound protein was eluted with 5 × 0.2 M glycine-HCl (pH 2.5) followed by 2 BV TBSA and combined eluates were immediately mixed with 10% v/v 3.0 M Tris-HCl, pH 8.5. The resin was then washed with 1 BV 3.0 M Tris-HCl and re-equilibrated with 10 BV TBSA. The purification was repeated a further two times, in each instance following reloading of the column with the used sample. Total antibody yield was estimated by spectrophotometric measurements at 280 nm to be 46 mg, using an estimated extinction coefficient of 1.8 mg^{-1} mL.cm^{-1}. The protein was further purified by SEC using a Sephadex S200 26/60 column (GE Lifesciences) equilibrated with 0.1 M NaCl containing 0.02% sodium azide from which it eluted as a single peak with size estimated at 200 kDa, based on BioRad Gel Filtration Standards. Coomassie stained SDS-PAGE (4–12% NuPAGE bis-Tris run in MOPS buffer, Life Technologies) revealed fractions containing a single 150 kDa band. Combined fractions provided a total of 34 mg.

The above-purified mAb 18–44 was coupled to Mini-leak Low divinyl sulfone-activated resin (Kem-En-Tec, Denmark) based on the manufacturer's instructions as follows: 15 mL (settled volume) Mini-leak Low activated resin was extensively washed with pure water in a 0.2 μm bottle filter. The damp resin was transferred to a 50 mL centrifuge tube (Thermo Fisher Scientific), followed by the addition of 12 mL pure water and 7.5 mL of coupling solution (30% polyethylene glycol 20,000 in 0.3 M $NaHCO_3$, pH 8.6) and was mixed before combining it with 2.8 mL of 0.1 M NaCl, 0.02% NaN_3 containing 34 mg Mab 18–44. The antibody was reacted with the resin at room temperature for 17 h with gentle mixing, after which the supernatant was shown to contain negligible amounts of the protein based on A280 spectrophotometric measurement. Unreacted divinyl sulfone groups were removed by addition of 30 mL 0.2 M ethanolamine-HCl, pH 9.0, for 2.5 h. The resin was cleaned by washing with 10 BV 0.2 M disodium hydrogen phosphate-sodium hydroxide (pH 11), then 10 BV 0.4 M trisodium citrate – HCl (pH 3) and it was extensively washed with, and then stored in, TBSA.

Protocols for using the above affinity resin for the purification of insulin receptor constructs were established using an in-house IR-B ectodomain construct termed "IR B17" (devoid of C-terminal leucine-zipper segments, detail not shown). IR B17 (0.5 mg in 2 mL) was bound to 0.5 mL resin equilibrated with TBSA in a 2 mL Poly-Prep column (Bio-Rad). The resin was washed with 10 BV TBSA. Upon elution, it was found that most of the IR B17 remained bound to the column at high concentrations (up to 5 mg mL^{-1}) of a 10-mer 18–44 epitope peptide TSPEEHRPFE (Prigent et al., 1990) (GenScript, USA); however, 0.4 M trisodium citrate-HCl, pH 3 buffer was effective in eluting the remaining material. Inclusion of salts to the eluent, such as 2 M $MgCl_2$ and lowering of the pH by means of a pH 5 sodium acetate-acetic acid buffer, improved the elution to acceptable levels.

Purification of HybZip

HybZip was purified typically from a single 5 L batch of conditioned medium to which was added PMSF (1:1000 dilution of 100 mM PMSF/propan-2-ol; Merck), sodium azide (Sigma-Aldrich) to 0.02% and 5 mL of 3 M Tris-HCl, pH 8.5 per litre of medium. The medium was then filtered through a 0.2 μm bottle filter (Thermo Scientific) to remove insoluble material. Sample volume reduction and concentration was achieved by cycling it continuously at room temperature through a stack of two Pellicon 3, 0.11 m^2, 10 kDa MW cut off concentrator cartridges (Merck-Millipore) until the sample was concentrated 10-fold. The concentrate was filtered through a 0.2 μm bottle filter and stored for use at 4°C or longer term at −20°C.

For purification, the filtered concentrate was run through a 20 mL BV, 50-mm diameter Sepharose CL-4B (GE Lifesciences) guard column to remove non-specifically adsorbing material. The hybrid receptor and IGF1-R homodimer were captured first on an in-house mAb 9E10 affinity resin column (eliminating in the process the IR-Bzip homodimer) before finally capturing the hybrid heterodimer on a 6-mL BV mAb 18-44 affinity column assembled as above. The use of the anti-c-myc mAb 9E10 (Hilpert et al., 2001) as an column affinity reagent has been described previously (McKern et al., 1997); such columns have been used in a number of our prior studies (Menting et al., 2013; Lawrence et al., 2016; Xu et al., 2020) and take advantage of the antibody's very high specificity. Unbound material containing the IGF1-R homodimer receptor was separated this way and the column was washed with at least ten column volumes (CV) of TBSA buffer. Hybrid receptor was eluted from the column with the 10-mer 18-44 peptide TSPEEHRPFE (GenScript, USA) as follows. 200 mg of the peptide was dissolved in 12 mL (2 CV) TBSA, which was used to suspend the mAb 18-44 resin, and dispense it into a 50 mL Falcon tube where it was placed on a rolling platform overnight at 4°C. The following day, the peptide eluate was drained and the beads were washed with an additional 12 mL of TBSA, which was then combined with the peptide eluate to give a total of 24 mL. Residual protein was eluted from the beads with 0.3 M tri-sodium citrate plus HCl buffer (pH 3.0) and retained for analysis. After re-equilibration of the beads, the concentrated receptor supernatant was cycled over the mAb 18-44 affinity column at least two further times or until all of the material had depleted as a result of capture. The peptide eluate was concentrated with an Ultra-15 30 kDa centrifugal concentrator (Merck-Millipore) and was purified further by size-exclusion chromatography (SEC) using a Superdex 200 10/300 GL column (GE Lifesciences) equilibrated with TBSA buffer (Figure S2B). The dimeric HybZip was separated from tetrameric species by performing further SEC runs through a pair of Superdex 200 10/300 GL columns coupled in tandem (Figures S2C and S2D), with the final product being assessed by SDS-PAGE analysis (Figure S2E).

Competition receptor binding assay

BALB/c3T3 overexpressing IGF-IR (P6) cells (Pietrzkowski et al., 1992) and R-IR-B cells (IGF-IR null mouse fibroblasts overexpressing the human insulin receptor isoform B; Denley et al., 2004) were cultured in DMEM, 10% fetal calf serum, 1% penicillin / streptomycin, G418 (250 $\mu g.mL^{-1}$). IGF-IR and IR-B were solubilized from the cells using lysis buffer (20 mM HEPES, 150 mM NaCl, 1.5 mM $MgCl_2$, 10% (v/v) glycerol, 1% (v/v) Triton X-100, 1 mM EGTA (pH 7.5)) for 1 h at 4°C and lysates were centrifuged for 10 min at 3500 rpm. Solubilized IGF-1R or IR-B (100 μL) or HybZip (0.25 μg) was used to coat each well of a white Greiner Lumitrac 600 plate previously coated with 24–31 anti-human IGF-1R antibody or 83-7 anti-human IR antibody (Soos et al., 1986, 1992). Antibodies were a kind gift from Prof. K. Siddle, University of Cambridge, UK. Europium-labelled IGF-I or insulin (~3,000,000 counts, measured using a Perkin Elmer Victor X4 2030 Multilabel Reader) was added to wells with increasing concentrations of competitive ligand IGF-I or insulin and incubated for 16 h at 4°C. Wells were washed three times with 20 mM Tris, 150 mM NaCl, 0.1% (v/v) Tween 20 and DELFIA enhancement solution (100 μL) was added. Time-resolved fluorescence was measured with 340-nm excitation and 612-nm emission filters on the same instrument. Replicate details are as follows: IGF-I binding IGF-1R, three assays with three replicates ($n = 9$; with three single individual measurements omitted as aberrant); IGF-I binding HybZip, four independent assays with three replicates each ($n = 12$); insulin binding IR-B, three independent assays with three replicates each ($n = 9$; with three single individual measurements omitted as aberrant); insulin binding HybZip, three independent assays with three replicates each ($n = 9$; with six individual measurement omitted as aberrant). Mean IC50 values were calculated with the statistical software package Prism

v9.0.0 (GraphPad Software) after curve fitting with non-linear regression (one-site) model. SEMs are shown for each data point (Figures S2F and S2G).

CryoEM data collection

Samples for cryoEM data collection were prepared as follows. Purified HybZip was concentrated to ca 0.2 mg.mL^{-1} (1 μM) in TBSA using a 0.5 mL 10 kDa Amicon Ultra concentrator and then combined in a 1:4 molar ratio (HybZip:IGF-I) with IGF-I ("Receptor grade"; Gropep, Australia; prepared at 10 mg.mL^{-1} in 10 mM HCl). All dataset were recorded on Cu 1.2/1.3 grids (Quantifoil Micro Tools GmbH) with mesh size of 200. Grids were glow discharged using 30 mA current for 30 s. Freeze plunging employed a Vitrobot (Thermo Fisher) set to humidity 100% and temperature 4°C. Prior to freeze plunging, the samples were applied to the glow-discharged grids which were subsequently blotted to achieve desired sample film thickness with a 3 s blot time and −3 blot force. Imaging employed a Titan Krios cryo-electron microscope (Thermo Fisher) equipped with a Gatan K2 Summit with Quantum-GIF energy filter, with a total of four data sets being collected. Imaging was performed in nanoprobe energy-filtered zero loss mode using a 20 eV slit width; data collection parameters for each of the four data sets are given in Table S1. A sample image and its Fourier transform are shown in Figures S3A and S3B, respectively.

Three-dimensional reconstruction

Data set 1 was patch-motion and patch-CTF corrected within cryoSPARC v2.11.0 (Punjani et al., 2017). All subsequent data processing steps were also performed with cryoSPARC. Images with thick ice and poor CTF fit were excluded, resulting in a retained set of 4723 images. A total 1674519 of particles were auto-picked using a template-free elliptical blob picking strategy. 2D classification resulted in 125999 particles being retained (from eight 2D selected classes), which were then subjected to ab initio 3D classification into two classes. The final subset comprised 88554 particles (Figure S3C). No particle classes appeared to reflect trace contaminant of tetrameric particles.

Data sets 2, 3 and 4 (comprising 1556, 5098 and 3210 movies, respectively) were patch-motion corrected and patch-CTF corrected within cryoSPARC v2.15.0 (Punjani et al., 2017). Images with thick ice and poor CTF fit were excluded, resulting in the retention of 1121, 4722, 2408 movies from the three respective data sets. Auto blob-picking, 2D classification and heterogeneous refinement were performed on each data set individually. The final number of particles retained for each of these data sets was 38938, 147375 and 118955, respectively, selected from nine, ten and eleven 2D classes, respectively No particle classes appeared to reflect trace contaminant of tetrameric particles.

The retained particles from the four data sets were then combined (Figure S3C) and subjected to 3D ab initio classification into three classes (Figure S3D). 160246 particles from one of these classes were then subjected to another round of 3D ab initio classification into three classes. Two of these classes (comprising 79641 and 71599 particles, respectively) were judged as similar and were combined for homogeneous refinement (resolution 4.06 Å) followed by local refinement (resolution 3.85 Å; Figure S3D). Examination of the resultant map revealed a structure that was closely similar to those determined prior for a single insulin in complex with IRΔβ.zip (Weis et al., 2018), for IGF-I in complex with holo-IGF-1R (Li et al., 2019) and for the closed form of IGF-II in complex with IGF-1R.zip (Xu et al., 2020), allowing ready docking of receptor domains into the map. The receptor monomers were distinguishable from each other by the respective presence (in IR) or absence (in IGF-1R) of the extended α-helical element within the sixth module of domain CR (Figure 1D).

Focused refinement then followed for the separate "head" and "leg" regions of the receptor (head: IGF-1R domains L1, CR, L2 and FnIII-1 plus IR domains L2, FnIII-1 and αCT plus IGF-I; legs: IGF-1R domains FnIII-2, FnIII-3 and ID plus IR domains L1, CR, FnIII-2, FnIII-3 and the remaining ID region outside of the "head" region), attaining resolution of 3.70 Å and 3.73 Å for "head" and "leg" regions, respectively (Figures S4A–S4E).

Model generation and real-space refinement

An initial model was generated by docking into the cryoEM map IR domains extracted from PDB entry 6PXV (Uchikawa et al., 2019), IGF-1R domains L1, CR and L2 extracted from PDB entry 6VWG (Xu et al., 2020), IGF-1R domains FnIII-1, FnIII-2 and FnIII-3 extracted from PDB entry 5U8R (Xu et al., 2018) and IGF-I extracted from 6PYH (Li et al., 2019); docking was performed using Chimera (Pettersen et al., 2004) followed by manual editing within Coot (Emsley et al., 2010). Initial real-space refinement was undertaken using phenix.real_space_refine (Afonine et al., 2018).

Further rebuilding of the model then proceeded as follows using ISOLDE (Croll, 2018). Residues from the IR and IGF-1R chains located in elements with defined secondary structure were restrained initially to the geometry of the corresponding sites in existing structures PDB 4ZXB (Croll et al., 2016), 5U8R (Xu et al., 2018) and 6VWG (Xu et al., 2020) using a combination of local distance and torsion restraints (Croll and Read, 2021). Each chain was then inspected from end to end, rebuilding as necessary and releasing restraints where the geometry of the applicable reference model clearly disagreed with the map. The reference restraints were then released and the model settled at 0 K, with further inspection and (where necessary and possible) correction of residual geometry outliers. The model was then refined in phenix.real_space_refine (Afonine et al., 2018), restraining torsions and atomic positions to the input geometry to reduce over-fitting to noisy, low-resolution regions. Final refinement employed the individual focus-refined maps within phenix.real_space_refine. Refinement statistics are provided in Table 1.

QUANTIFICATION AND STATISTICAL ANALYSIS

Receptor competition binding assay data were analysed using Prism v9.0.0 (GraphPad Software). Details of the measurements, number of replicates and statistical reporting can be found in the Method details section of the STAR methods. The number of points (*n*) measured per experiment and the number of replicates chosen were based on that being sufficient to allow quantitative assessment of the relative affinity of HybZip, holo-IGF-1R and holo-IR-B for ligand. The results are reported in-line in the text (Results subsection Characterization of HybZip) and include mean values accompanied by 95% confidence intervals. Overlap of respective 95% confidence intervals was judged sufficient to conclude that the affinities were similar.

Structure, Volume *30*

Supplemental Information

How insulin-like growth factor I binds to a hybrid insulin receptor type 1 insulin-like growth factor receptor

Yibin Xu, Mai B. Margetts, Hari Venugopal, John G. Menting, Nicholas S. Kirk, Tristan I. Croll, Carlie Delaine, Briony E. Forbes, and Michael C. Lawrence

A **Protein Sequence of IR-Bzip**

1 HLYPGEVCPGMDIRNNLTRLHELENCSVIEGHLQILLMFKTRPEDFRDLSFPKLIMITDYLLLFRVYGLESLKDLFPNLT 80
81 VIRGSRLFFNYALVIFEMVHLKELGLYNLMNITRGSVRIEKNNELCYLATIDWSRILDSVEDNYIVLNKDDNEECGDICP 160
161 GTAKGKTNCPATVINGQFVERCWTHSHCQKVCPTICKSHGCTAEGLCCHSECLGNCSQPDDPTKCVACRNFYLDGRCVET 240
241 CPPPYYHFQDWRCVNFSFCQDLHHKCKNSRRQGCHQYVIHNNKCIPECPSGYTMNSSNLLCTPCLGPCPKVCHLLEGEKT 320
321 IDSVTSAQELRGCTVINGSLIINIRGGNNLAAELEANLGLIEEISGYLKIRRSYALVSLSFFRKLRLIRGETLEIGNYSF 400
401 YALDNQNLRQLWDWSKHNLTITQGKLFFHYNPKLCLSEIHKMEEVSGTKGRQERNDIALKTNGDQASCENELLKFSYIRT 480
481 SFDKILLRWEPYWPPDFRDLLGFMLFYKEAPYQNVTEFDGQDACGSNSWTVVDIDPPLRSNDPKSQNHPGWLMRGLKPWT 560
561 QYAIFVKTLVTFSDERRTYGAKSDIIYVQTDATNPSVPLDPISVSNSSSQIILKWKPPSDPNGNITHYLVFWERQAEDSE 640
641 LFELDYCLKGLKLPSRTWSPPFESEDSQKHNQSEYEDSAGECCSCPKTDSQILKELEESSFRKTFEDYLHNVVFVPRKTS 720
721 SGTGAEDPRPSRKRRSLGDVGNVTVAVPTVAAFPNTSSTSVPTSPEEHRPFEKVVNKESLVISGLRHFTGYRIELQACNQ 800
801 DTPEERCSVAAYVSARTMPEAKADDIVGPVTHEIFENNVVHLMWQEPKEPNGLIVLYEVSYRRYGDEELHLCVSRKHFAL 880
881 ERGCRLRGLSPGNYSVRIRATSLAGNGSWTEPTYFYVTDYLDVPSNIARMKQLEDKVEELLSKNYHLENEVARLKKLVGE 960
961 R

B **Protein Sequence of IGF-1Rzip**

1 EICGPGIDIRNDYQQLKRLENCTVIEGYLHILLISKAEDYRSYRFPKLTVITEYLLLFRVAGLESLGDLFPNLTVIRGWK 80
81 LFYNYALVIFEMTNLKDIGLYNLRNITRGAIRIEKNADLCYLSTVDWSLILDAVSNNYIVGNKPPKECGDLCPGTMEEKP 160
161 MCEKTTINNEYNYRCWTTNRCQKMCPSTCGKRACTENNECCHPECLGSCSAPDNDTACVACRHYYYAGVCVPACPPNTYR 240
241 FEGWRCVDRDFCANILSAESSDSEGFVIHDGECMQECPSGFIRNGSQSMYCIPCEGPCPKVCEEEKKTKTIDSVTSAQML 320
321 QGCTIFKGNLLINIRRGNNIASELENFMGLIEVVTGYVKIRHSHALVSLSFLKNLRLILGEEQLEGNYSFYVLDNQNLQQ 400
401 LWDWDHRNLTIKAGKMYFAFNPKLCVSEIYRMEEVTGTKGRQSKGDINTRNNGERASCESDVLHFTSTTTSKNRIIITWH 480
481 RYRPPDYRDLISFTVYYKEAPFKNVTEYDGQDACGSNSWNMVDVDLPPNKDVEPGILLHGLKPWTQYAVYVKAVTLTMVE 560
561 NDHIRGAKSEILYIRTNASVPSIPLDVLSASNSSSQLIVKWNPPSLPNGNLSYYIVRWQRQPQDGYLYRHNYCSKDKIPI 640
641 RKYADGTIDIEEVTENPKTEVCGGEKGPCCACPKTEAEKQAEKEEAEYRKVFENFLHNSIFVPRPERKRRDVMQVANTTM 720
721 SSRSRNTTAADTYNITDPEELETEYPFFESRVDNKERTVISNLRPFTLYRIDIHSCNHEAEKLGCSASNFVFARTMPAEG 800
801 ADDIPGPVTWEPRPENSIFLKWPEPENPNGLILMYEIKYGSQVEDQRECVSRQEYRKYGGAKLNRLNPGNYTARIQATSL 880
881 SGNGSWTDPVFFYVQAKTGYENFIHRMKQLEDKVEELLSKNYHLENEVARLKKLVGERSSSEQKLISEEDLN 952

Figure S1. Protein sequences of the two constructs use to form HybZip, Related to STAR Methods.

(**A**) IR-Bzip, comprising residues 1-928 of the human IR-B isoform followed by the 33-residue GCN4 zipper sequence (*orange*).

(**B**) IGF-1Rzip, comprising residues 1-905 of human IGF-1R followed by the 33-residue GCN4 zipper sequence (*orange*) followed by a three-serine spacer and the c-myc tag sequence EQKLISEEDLN (*blue*).

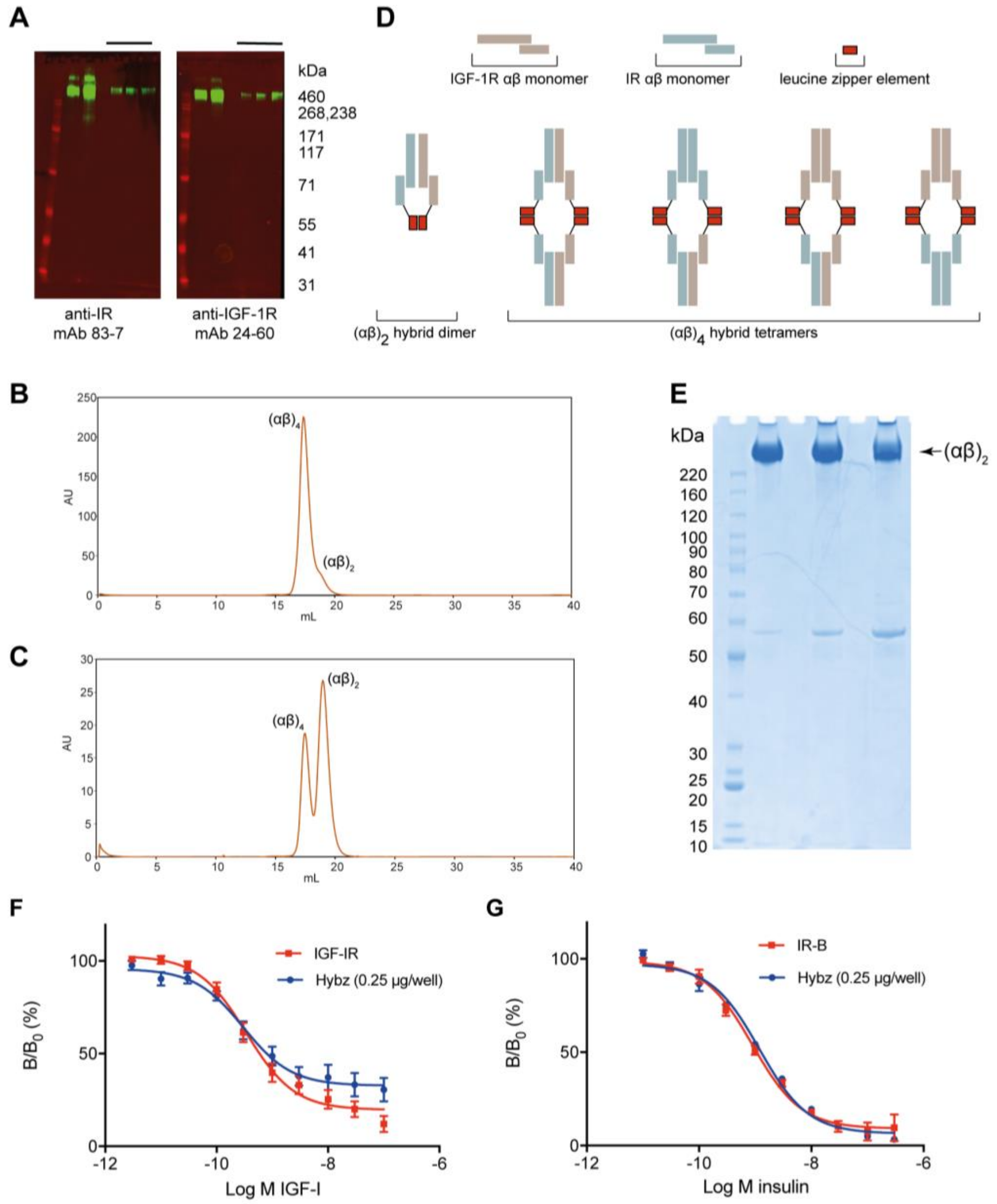

Figure S2. Purification and characterization of HybZip, Related to STAR Methods.

(**A**) Western blots of protein product obtained after serial elution from both 9E10 mAb and 18-44 mAb beads, showing presence of both species in the material (lanes with *bar* on right-hand side of each blot). Left-hand lanes in each blot correspond to pH 3 eluted material obtained from final column wash. Superfluous boundary has been removed from both blot images.

(**B**) Size-exclusion chromatogram obtained from protein product post affinity purification. The dominant peak corresponds to $(\alpha\beta)_4$ species, with a shoulder corresponding to $(\alpha\beta)_2$ species.

(**C**) Enrichment of $(\alpha\beta)_2$ species following re-run of pooled shoulder fractions from (B).

(**D**) Schematic illustrating leucine-zipper cross-linking of receptor ectodomain dimers to form hybrid tetrameric species. All four tetrameric species will be affinity co-purified with the desired hybrid dimer, but can be separated from it by size-exclusion chromatography (see panels B and C).

(**E**) Non-reducing SDS-PAGE gel of pooled $(\alpha\beta)_2$ fractions depicted in (C). Superfluous boundary and blank lanes (to the right) have been removed from the gel image.

(**F**) Competitive displacement assay of labelled IGF-I bound to holo-IGF-1R (n = 9) and to HybZip (n = 12) by unlabelled IGF-I. Error bars reflect standard error of the mean and are omitted when smaller than marker size. B/B_0 = percentage of binding in the absence of competing ligand.

(**G**) Competitive displacement assay of labelled insulin bound to holo-IR-B (n = 9) and to HybZip (n = 9) by unlabelled insulin. Error bars reflect standard error of the mean and are omitted when smaller than marker size. B/B_0 = percentage of binding in the absence of competing ligand.

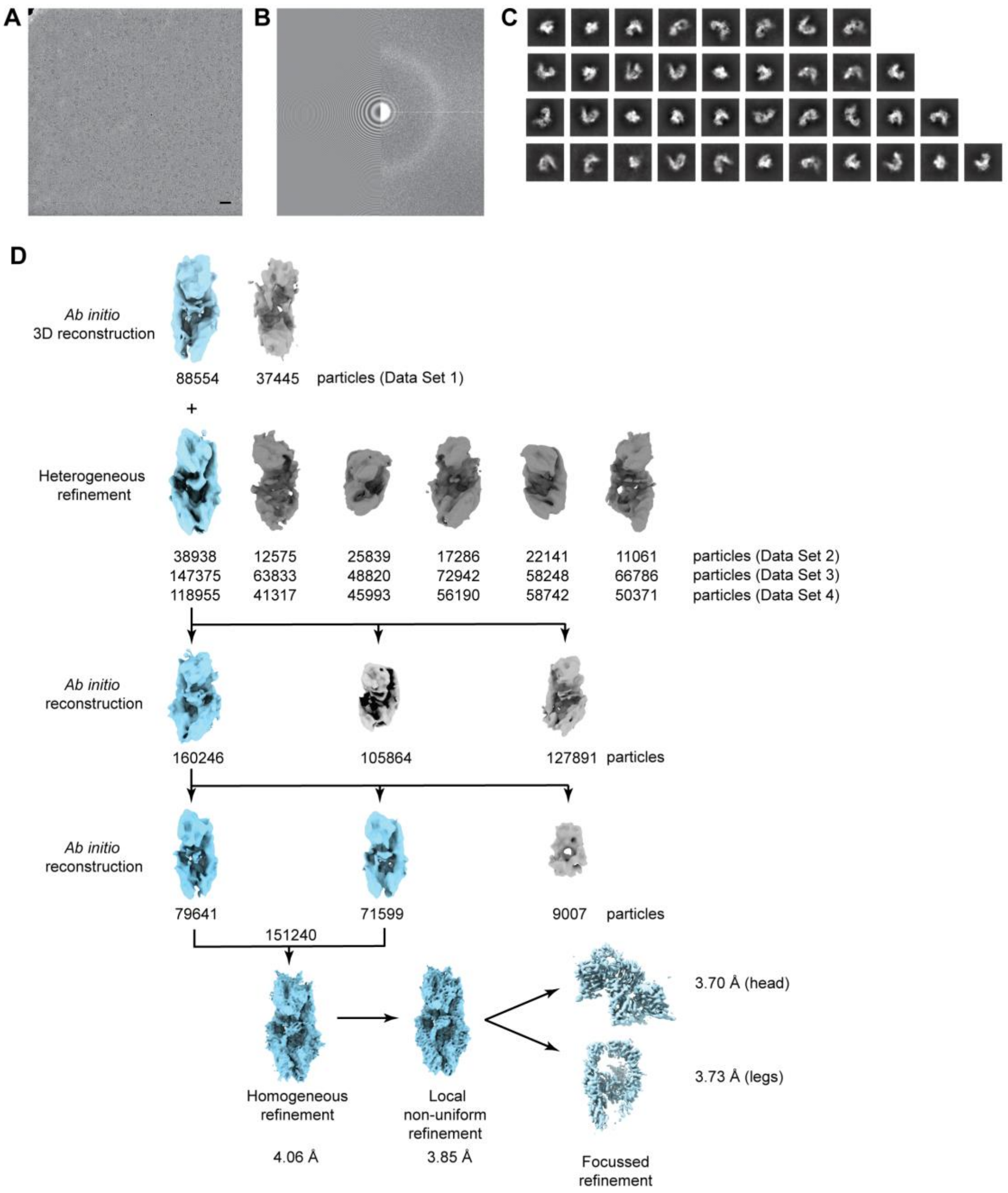

Figure S3. CryoEM reconstruction of IGF-I-complexed HybZip, Related to STAR Methods.

(**A**) Representative image obtained after motion correction (scale bar = 200 Å)

(**B**) Contrast transfer function associated with (A).

(**C**) 2D classes of particles selected from the four data sets (one data set per row; individual class images have been cropped to facilitate enlarged display).

(**D**) Flowchart showing the process of 3D reconstruction of IGF-I-complexed HybZip.

Full details are provided in the **STAR Methods Detail** section.

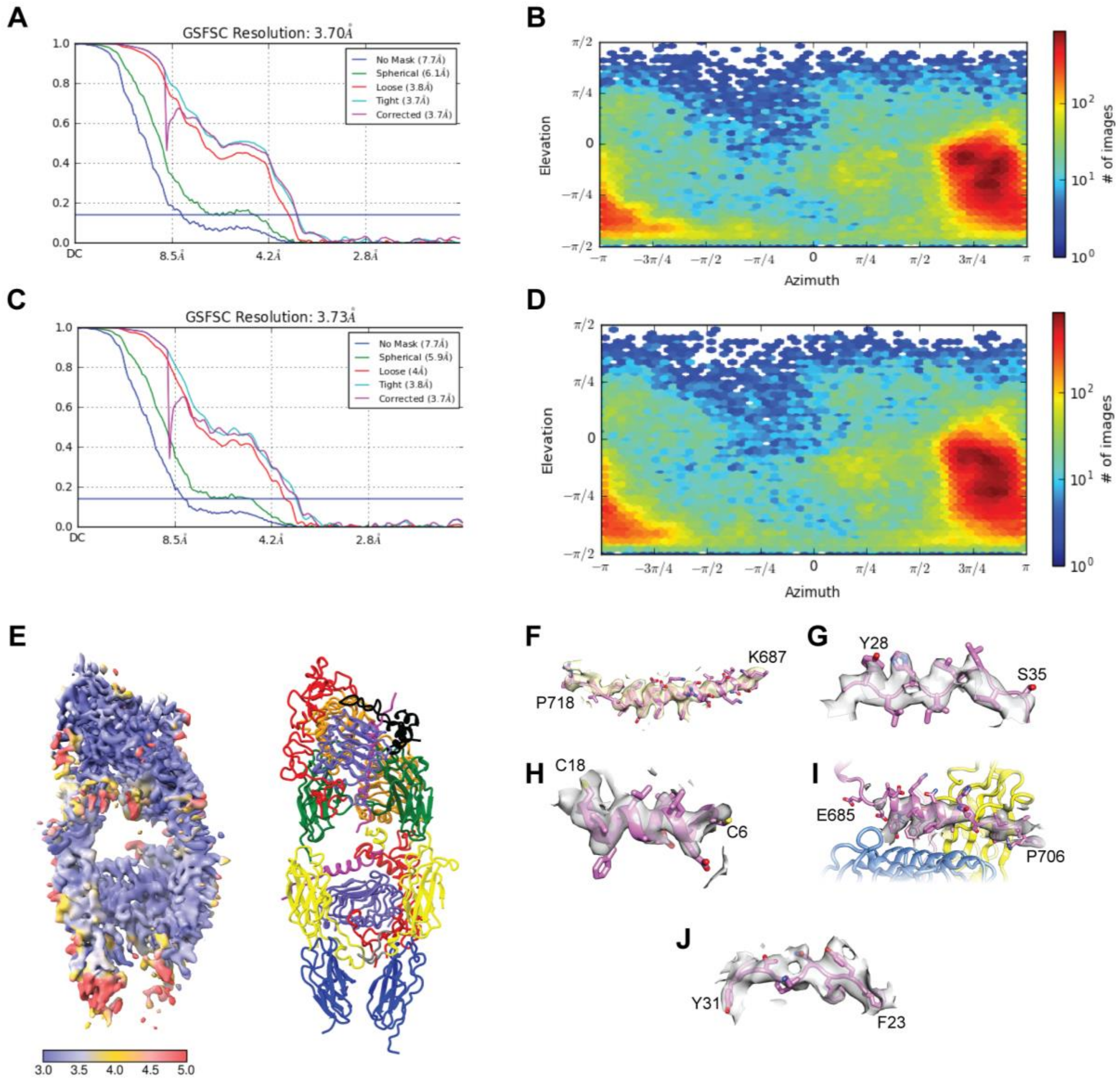

Figure S4. Quality of the 3D reconstructions of the two receptor volumes, Related to STAR Methods.

(**A**),(**C**) Gold-standard Fourier shell correlation (GSFSC) plots for the half-maps associated with the reconstruction of the "head" and "leg" region, respectively, of the IGF-I-complexed HybZip.

(**B**), (**D**) Angular distribution of particles contributing to the reconstruction of the "head" and "leg" region, respectively, of the IGF-I-complexed HybZip.

(**E**) Left panel: overlay of the "head" and "leg" regions maps, colored according to the local resolution. Right panel: ribbon diagram of IGF-I-complexed HybZip, oriented and scaled to match the local-resolution map in the left panel.

(**F**) CryoEM potential density for IR αCT segment, residues Lys687-Pro718. Contour level 0.201, display restricted to within 3.0 Å of depicted residues.

(**G**) CryoEM potential density for IGF-1R domain L1, residues Tyr28-Ser35. Contour level 0.230, display restricted to within 3.0 Å of depicted residues.

(**H**) CryoEM potential density for B domain helix of IGF-I, residues Cys6-Cys18. Contour level 0.242, display restricted to within 3.0 Å of depicted residues.

(**I**) CryoEM potential density for IGF-1R αCT segment, residues Glu685-Pro706. Contour level 0.201, display restricted to within 3.4 Å of depicted residues.

(**J**) CryoEM potential density for C domain of IGF-I, residues Phe23-Tyr31. Contour level 0.160, display restricted to within 3.0 Å of depicted residues.

Table S1. CryoEM data collection parameters, related to STAR METHODS

Data Set:	1	2	3	4
Pixel size (Å)	1.06	1.06	1.06	1.06
Dose rate ($e^{-}.pix^{-1}.sec^{-1}$)	8	6	6	6
Total dose ($e^{-}.Å^{-2}$)	50	52	52	52
No. frames/movie	36	50	50	50
C_s	2.7	2.7	2.7	2.7
kV	300	300	300	300
